# Weighted analysis of general microarray experiments

**DOI:** 10.1186/1471-2105-8-387

**Published:** 2007-10-15

**Authors:** Anders Sjögren, Erik Kristiansson, Mats Rudemo, Olle Nerman

**Affiliations:** 1 Mathematical Statistics, Chalmers University of Technology, 412 96 Göteborg, Sweden; 2 Mathematical Statistics, Göteborg University, 412 96 Göteborg, Sweden

## Abstract

**Background:**

In DNA microarray experiments, measurements from different biological samples are often assumed to be independent and to have identical variance. For many datasets these assumptions have been shown to be invalid and typically lead to too optimistic p-values. A method called WAME has been proposed where a variance is estimated for each sample and a covariance is estimated for each pair of samples. The current version of WAME is, however, limited to experiments with paired design, e.g. two-channel microarrays.

**Results:**

The WAME procedure is extended to general microarray experiments, making it capable of handling both one- and two-channel datasets. Two public one-channel datasets are analysed and WAME detects both unequal variances and correlations. WAME is compared to other common methods: fold-change ranking, ordinary linear model with t-tests, LIMMA and weighted LIMMA. The p-value distributions are shown to differ greatly between the examined methods. In a resampling-based simulation study, the p-values generated by WAME are found to be substantially more correct than the alternatives when a relatively small proportion of the genes is regulated. WAME is also shown to have higher power than the other methods. WAME is available as an R-package.

**Conclusion:**

The WAME procedure is generalized and the limitation to paired-design microarray datasets is removed. The examined other methods produce invalid p-values in many cases, while WAME is shown to produce essentially valid p-values when a relatively small proportion of genes is regulated. WAME is also shown to have higher power than the examined alternative methods.

## Background

The DNA microarray technique involves a series of steps, from the harvesting of cells or biopsies to the preprocessing of the scanned arrays, before analysable data are obtained. During several of these steps the quality can be affected by random factors. For instance, depending on the handling of a biological sample the mRNA can be more or less degraded [1], and the cell-type composition of a biopsy can be more or less representative for the tissue in question. When arrays share sources of variation the deviations from the nominal value will be correlated. For example, two arrays from sources with degraded RNA will both tend to underestimate the expression of easily degradable genes, and two biopsies with a similar and non-representative cell-type composition will deviate in a similar fashion from the average expression for the ideal cell-type composition.

The procedure *Weighted Analysis of Microarray Experiments *(WAME) [2,3] introduced a model where a covariance-structure matrix common for all genes aims at catching differences in quality by differences in variances and covarying deviations by correlations between arrays. For computations of test statistics and estimators this resulted in weighting of observations according to the estimated covariance-structure matrix, giving lower weight to imprecise or positively correlated arrays.

In order for the estimation of the covariance matrix to work in the current WAME method, the measurements of most genes must only measure noise, i.e. have an expected value of zero. This is the case in experiments where pair-wise log-ratios are observed and where few genes are differentially expressed between any of the pairwise measured conditions. In the present paper, this crucial constraint will be relaxed to only require that most genes are non-differentially expressed between the conditions actually being compared. Thus, non-paired experiments can be analysed, e.g. many additional ones based on one-channel microarray data. The relaxation is realised by transforming the data to remove irrelevant information in a manner yielding transformed data with expectation zero for non-differentially expressed genes, after which the current WAME method is applied. The transformed data are shown to give equivalent tests and estimates to those of the original data, given the corresponding covariance-structure matrices.

### Problem formulation and current methods

Given a microarray experiment with *n *arrays and *m *genes, we observe for each gene *g *an *n*-dimensional vector **X**_*g *_of log_2 _transformed values measuring mRNA abundance. In WAME the vector **X**_***g ***_is assumed to have expectation ***μ***_***g ***_described by a design matrix *D *and a gene-specific parameter vector ***γ***_***g***_, typically having one dimension per studied condition. A covariance-structure matrix Σ, common for all genes, is used to model differences in quality between arrays as different variances and shared sources of variation between arrays as correlations. A gene-specific variance-scaling factor *c*_*g *_is assumed to have inverse gamma prior distribution with a global shape parameter *α*. Conditional on *c*_*g *_the vector **X**_***g ***_is assumed to have a normal distribution with covariance matrix *c*_*g*_Σ. A matrix *C *specifies the differential expression vector ***δ***_*g*_, describing the linear combinations of the parameters that are of main interest. Formally,

μg=Dγg,Xg|cg~N(μg,cgΣ),cg~Γ−1(α,1),
 MathType@MTEF@5@5@+=feaafiart1ev1aaatCvAUfKttLearuWrP9MDH5MBPbIqV92AaeXatLxBI9gBaebbnrfifHhDYfgasaacH8akY=wiFfYdH8Gipec8Eeeu0xXdbba9frFj0=OqFfea0dXdd9vqai=hGuQ8kuc9pgc9s8qqaq=dirpe0xb9q8qiLsFr0=vr0=vr0dc8meaabaqaciaacaGaaeqabaqabeGadaaakeaafaqadeWadaaabaaccmGae8hVd02aaSbaaSqaaiabdEgaNbqabaaakeaacqGH9aqpaeaacqWGebarcqWFZoWzdaWgaaWcbaGaem4zaCgabeaakiabcYcaSaqaaGqabiab+HfaynaaBaaaleaacqWGNbWzaeqaaOGaeiiFaWNaem4yam2aaSbaaSqaaiabdEgaNbqabaaakeaacqGG+bGFaeaacqqGobGtcqGGOaakcqWF8oqBdaWgaaWcbaGaem4zaCgabeaakiabcYcaSiabdogaJnaaBaaaleaacqWGNbWzaeqaaOGaeu4OdmLaeiykaKIaeiilaWcabaGaem4yam2aaSbaaSqaaiabdEgaNbqabaaakeaacqGG+bGFaeaacqqHtoWrdaahaaWcbeqaaiabgkHiTiabigdaXaaakiabcIcaOGGaciab9f7aHjabcYcaSiabigdaXiabcMcaPiabcYcaSaaaaaa@58F6@

and variables corresponding to different genes are assumed independent. We want to estimate the differential expression

***δ***_***g ***_= *C**γ***_***g***_

or we want to test for differential expression

H0:δg=0HA:δg≠0.
 MathType@MTEF@5@5@+=feaafiart1ev1aaatCvAUfKttLearuWrP9MDH5MBPbIqV92AaeXatLxBI9gBaebbnrfifHhDYfgasaacH8akY=wiFfYdH8Gipec8Eeeu0xXdbba9frFj0=OqFfea0dXdd9vqai=hGuQ8kuc9pgc9s8qqaq=dirpe0xb9q8qiLsFr0=vr0=vr0dc8meaabaqaciaacaGaaeqabaqabeGadaaakeaafaqadeGadaaabaGaemisaG0aaSbaaSqaaiabicdaWaqabaGccqGG6aGoiiWacqWF0oazdaWgaaWcbaGaem4zaCgabeaaaOqaaiabg2da9aqaaGqabiab+bdaWaqaaiabdIeainaaBaaaleaacqWGbbqqaeqaaOGaeiOoaOJae8hTdq2aaSbaaSqaaiabdEgaNbqabaaakeaacqGHGjsUaeaacqGFWaamcqGGUaGlaaaaaa@3F3A@

In the current version of WAME [2,3] the estimation of the covariance-structure matrix Σ is based on a temporary assumption of expectation zero, ***μ***_***g ***_= **0**, for all genes, which is shown to give reasonable results if the expectation is close to zero for most genes. Thus, this is a suitable assumption for data with paired observations and few regulated genes between the pair-wise measured conditions.

The WAME model can be compared with the ordinary linear model (OLM) [4],

**X**_***g ***_~ N(***μ***_***g***_, *c*_*g*_*I*)

which gives rise to the ordinary t- or F-tests, and with a widely used empirical Bayes model proposed in [5] and implemented in the LIMMA package [6],

Xg|cg~N(μg,cgI),cg~Γ−1(α,β).
 MathType@MTEF@5@5@+=feaafiart1ev1aaatCvAUfKttLearuWrP9MDH5MBPbIqV92AaeXatLxBI9gBaebbnrfifHhDYfgasaacH8akY=wiFfYdH8Gipec8Eeeu0xXdbba9frFj0=OqFfea0dXdd9vqai=hGuQ8kuc9pgc9s8qqaq=dirpe0xb9q8qiLsFr0=vr0=vr0dc8meaabaqaciaacaGaaeqabaqabeGadaaakeaafaqadeGadaaabaacbeGae8hwaG1aaSbaaSqaaiabdEgaNbqabaGccqGG8baFcqWGJbWydaWgaaWcbaGaem4zaCgabeaaaOqaaiabc6ha+bqaaiabb6eaojabcIcaOGGadiab+X7aTnaaBaaaleaacqWGNbWzaeqaaOGaeiilaWIaem4yam2aaSbaaSqaaiabdEgaNbqabaGccqWGjbqscqGGPaqkcqGGSaalaeaacqWGJbWydaWgaaWcbaGaem4zaCgabeaaaOqaaiabc6ha+bqaaiabfo5ahnaaCaaaleqabaGaeyOeI0IaeGymaedaaOGaeiikaGccciGae0xSdeMaeiilaWIae0NSdiMaeiykaKIaeiOla4caaaaa@4FD3@

The novel feature of WAME was thus the introduction of the quality modelling covariance-structure matrix Σ.

After the introduction of WAME, a weighted version of LIMMA was proposed [7], which we will refer to as wLIMMA. There, a model with array-wise variance scales but no correlations is used,

Xg|cg~N(μg,cgdiag(σ12,...,σn2)),cg~Γ−1(α,β).
 MathType@MTEF@5@5@+=feaafiart1ev1aaatCvAUfKttLearuWrP9MDH5MBPbIqV92AaeXatLxBI9gBaebbnrfifHhDYfgasaacH8akY=wiFfYdH8Gipec8Eeeu0xXdbba9frFj0=OqFfea0dXdd9vqai=hGuQ8kuc9pgc9s8qqaq=dirpe0xb9q8qiLsFr0=vr0=vr0dc8meaabaqaciaacaGaaeqabaqabeGadaaakeaafaqadeGadaaabaacbeGae8hwaG1aaSbaaSqaaiabdEgaNbqabaGccqGG8baFcqWGJbWydaWgaaWcbaGaem4zaCgabeaaaOqaaiabc6ha+bqaaiabb6eaojabcIcaOGGadiab+X7aTnaaBaaaleaacqWGNbWzaeqaaOGaeiilaWIaem4yam2aaSbaaSqaaiabdEgaNbqabaGccqqGKbazcqqGPbqAcqqGHbqycqqGNbWzcqGGOaakiiGacqqFdpWCdaqhaaWcbaGaeGymaedabaGaeGOmaidaaOGaeiilaWIaeiOla4IaeiOla4IaeiOla4IaeiilaWIae03Wdm3aa0baaSqaaiabd6gaUbqaaiabikdaYaaakiabcMcaPiabcMcaPiabcYcaSaqaaiabdogaJnaaBaaaleaacqWGNbWzaeqaaaGcbaGaeiOFa4habaGaeu4KdC0aaWbaaSqabeaacqGHsislcqaIXaqmaaGccqGGOaakcqqFXoqycqGGSaalcqqFYoGycqGGPaqkcqGGUaGlaaaaaa@623B@

The parameters are estimated using a restricted maximum-likelihood (REML) approach.

A widely used approach is to only consider the ordinary least-squares estimated differential expression, often referred to as the log fold-change, here abbreviated as FC, or as the average M-value. In the present paper, the ranking of the genes imposed by this method will be included in comparisons, when applicable.

## Results

### The new version of WAME

In the current version of WAME [2,3] the covariance-structure matrix Σ is estimated using a temporary assumption that ***μ***_***g ***_= **0 **for most genes, i.e. that the measurements of most genes consist solely of biological and technical noise. In the new version of WAME we relax this to only assume that most genes are non-differentially expressed, i.e. ***δ***_***g ***_= **0**. This allows a much larger class of experimental designs and design matrices *D*, most notably unpaired designs.

The trick used is to transform the data and consider

Yg=Xg−μ˜g0
 MathType@MTEF@5@5@+=feaafiart1ev1aaatCvAUfKttLearuWrP9MDH5MBPbIqV92AaeXatLxBI9gBaebbnrfifHhDYfgasaacH8akY=wiFfYdH8Gipec8Eeeu0xXdbba9frFj0=OqFfea0dXdd9vqai=hGuQ8kuc9pgc9s8qqaq=dirpe0xb9q8qiLsFr0=vr0=vr0dc8meaabaqaciaacaGaaeqabaqabeGadaaakeaaieqacqWFzbqwdaWgaaWcbaGaem4zaCgabeaakiabg2da9iab=HfaynaaBaaaleaacqWGNbWzaeqaaOGaeyOeI0cccmGaf4hVd0MbaGaadaqhaaWcbaGaem4zaCgabaGaeGimaadaaaaa@386D@

where μ˜g0
 MathType@MTEF@5@5@+=feaafiart1ev1aaatCvAUfKttLearuWrP9MDH5MBPbIqV92AaeXatLxBI9gBaebbnrfifHhDYfgasaacH8akY=wiFfYdH8Gipec8Eeeu0xXdbba9frFj0=OqFfea0dXdd9vqai=hGuQ8kuc9pgc9s8qqaq=dirpe0xb9q8qiLsFr0=vr0=vr0dc8meaabaqaciaacaGaaeqabaqabeGadaaakeaaiiWacuWF8oqBgaacamaaDaaaleaacqWGNbWzaeaacqaIWaamaaaaaa@30EB@ is a suitable linear estimator of ***μ***_***g ***_which is unbiased under *H*_0 _and which preserves the estimability of the differential expression ***δ***_***g***_, based on only the transformed data (see Methods for details). An example is (8) below where for each gene the mean value of all arrays is subtracted.

Since the transformed data contain only noise for non-differentially expressed genes by construction, the current version of WAME can essentially be applied to the transformed data **Y**_***g***_. As before, the covariance-structure matrix (now Σ_*Y*_) and the hyperparameter *α *are first estimated under a provisional assumption (now ***δ***_***g ***_= **0**). The maximum likelihood estimates of ***δ***_***g ***_and the likelihood ratio test statistics of (3) are then computed. The tests and estimators are in fact unchanged by the transformation (7), if the covariance-structure matrices for the transformed and untransformed data are known (details given in Methods). WAME is implemented as a package for the R language [8] and is available at [9].

### Evaluation on real and resampled data

To investigate the properties of the new version of WAME, two real datasets are examined. Briefly, they are analysed both using WAME and the current methods described in Background. Array-specific weights, p-value distributions and rankings are produced showing clear differences between the procedures, most notably in the p-value distributions. To investigate the power of the different procedures and to look at p-value distributions in a controlled but realistic setting, we also analyse simulated data with real noise from the studied datasets and synthetic signal.

#### Description of the real datasets

Two public one-channel microarray datasets are analysed. The datasets are selected from the NCBI GEO database [10] with the criteria of having unpaired design and being sufficiently large to allow for the resample-based simulations in Resampled data below.

In the first dataset [11], biopsies were taken from the left atrium from 20 human hearts with normal sinus rythm and 10 hearts with permanent atrial fibrillation. It is here referred to as Atrium. In the second dataset [12], mechanisms in chronic obstructive pulmonary disease, COPD, were investigated by taking lung tissue biopsies from 12 smokers with mild or no emphysema and from 18 smokers with severe emphysema. In both datasets one Affymetrix HGU-133A array was used for each patient. In the present paper RMA [13] is used to obtain expression measures from the raw probe-wise intensities. The analyses are performed using the R language and the Bioconductor framework [14].

#### Analysis of the real datasets

A natural parameterisation of the included datasets is to have one parameter per condition, yielding design and hypothesis matrices

D=[10⋮⋮1001⋮⋮01] and C=[−11].
 MathType@MTEF@5@5@+=feaafiart1ev1aaatCvAUfKttLearuWrP9MDH5MBPbIqV92AaeXatLxBI9gBaebbnrfifHhDYfgasaacH8akY=wiFfYdH8Gipec8Eeeu0xXdbba9frFj0=OqFfea0dXdd9vqai=hGuQ8kuc9pgc9s8qqaq=dirpe0xb9q8qiLsFr0=vr0=vr0dc8meaabaqaciaacaGaaeqabaqabeGadaaakeaacqWGebarcqGH9aqpdaWadaqaauaabeqagiaaaaqaaiabigdaXaqaaiabicdaWaqaaiabl6Uinbqaaiabl6UinbqaaiabigdaXaqaaiabicdaWaqaaiabicdaWaqaaiabigdaXaqaaiabl6Uinbqaaiabl6UinbqaaiabicdaWaqaaiabigdaXaaaaiaawUfacaGLDbaacqqGGaaicqqGHbqycqqGUbGBcqqGKbazcqqGGaaicqWGdbWqcqGH9aqpdaWadaqaauaabeqabiaaaeaacqGHsislcqaIXaqmaeaacqaIXaqmaaaacaGLBbGaayzxaaGaeiOla4caaa@4D4F@

Under the null hypothesis, for each gene *g *and array *i*, an unbiased estimator of the expected value of the measurement *X*_*ig *_is obtained by the gene-wise mean value over all arrays from both groups. The transformation then becomes a subtraction of that mean value, cf. (7),

Yig=Xig−1n∑j=1nXjg.
 MathType@MTEF@5@5@+=feaafiart1ev1aaatCvAUfKttLearuWrP9MDH5MBPbIqV92AaeXatLxBI9gBaebbnrfifHhDYfgasaacH8akY=wiFfYdH8Gipec8Eeeu0xXdbba9frFj0=OqFfea0dXdd9vqai=hGuQ8kuc9pgc9s8qqaq=dirpe0xb9q8qiLsFr0=vr0=vr0dc8meaabaqaciaacaGaaeqabaqabeGadaaakeaacqWGzbqwdaWgaaWcbaGaemyAaKMaem4zaCgabeaakiabg2da9iabdIfaynaaBaaaleaacqWGPbqAcqWGNbWzaeqaaOGaeyOeI0YaaSaaaeaacqaIXaqmaeaacqWGUbGBaaWaaabCaeaacqWGybawdaWgaaWcbaGaemOAaOMaem4zaCgabeaaaeaacqWGQbGAcqGH9aqpcqaIXaqmaeaacqWGUbGBa0GaeyyeIuoakiabc6caUaaa@453E@

Note how the transformation preserves the difference in mean value between the two groups of arrays.

**Figure 1:**
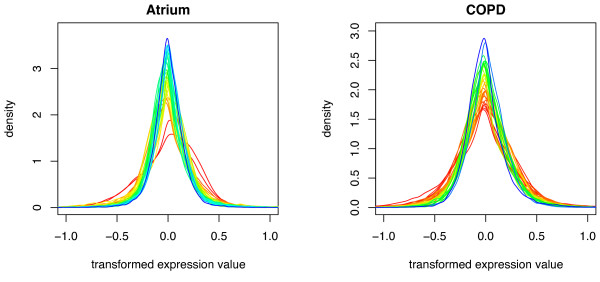
**Density plots**. Distribution of transformed expression values, **Y**, for the different arrays, in the two datasets. Colour-coding according to sample variance is used for increased clarity (blue for low variance, red for high variance). Differences in variability can be noted for both datasets.

If the elements in **X**_***g ***_from the different arrays had in fact independent and identically distributed noise for each fixed gene *g *as assumed in OLM and unweighted LIMMA, the noise in **Y**_***g ***_would have equal variances for all arrays. In Figure 1 array-wise density estimates for the transformed expression values are shown. For arrays from the same condition the distributions should be identical, reflecting the combined variability of signal and noise. For unregulated genes the expectation of **Y**_***g ***_is zero, so if the assumption of few regulated genes holds the densities from all arrays should furthermore be essentially equal. Examination of Figure 1 reveals that neither of these statements are true, indicating that some variances are highly unequal.

**Figure 2:**
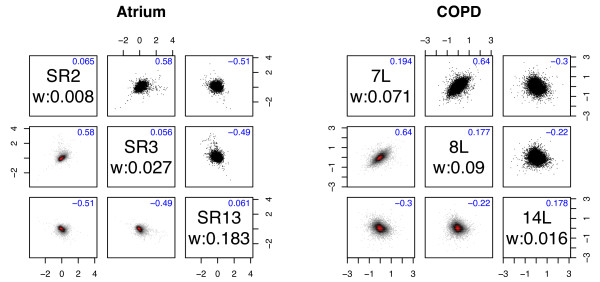
**Pairwise plots**. Transformed expression values, **Y**_*g*_, for selected pairs of arrays within the same group. Different pairs within the same group have distinctly different correlations. Upper triangle contains scatterplots. Lower triangle contains heatmaps of the corresponding two-dimensional kernel density estimates, where the majority of the genes are in the red portion of the plot, revealing important trends inside the black clouds. Diagonal red clouds in the heat maps reveal correlations between arrays. Off-diagonal numbers show estimated correlations from WAME. Diagonal boxes contain sample names and weights as well as estimated variances from WAME.

Analogously, all pairs of arrays within each condition should have a common joint distribution and when few genes are regulated all pairs of arrays should essentially have a common joint distribution with a small negative correlation of -1/(*n *- 1). Examination of scatter plots for all pairs of arrays shows that this is clearly not the case (some obvious examples are shown in Figure 2, all pairs are included in Additional file 1 and Additional file 2).

As expected from the observations above, unequal variances and non-zero correlations are estimated in the analyses with WAME, giving rise to highly unequal weights in the estimates of the differential expressions (shown in Table 1 and Table 2). In fact, the sign of the weight for some arrays even get switched compared to the sign of the weight of the other arrays from the same condition. This is an effect of strong correlations combined with unequal variances. It is an issue which is further addressed in Discussion.

**Table 1:** WAME weights for the Atrium dataset. Weights in percent from estimate of differential expression using WAME on the Atrium dataset.

Sinus rythm							Atrial fibrillation			
	
3.0	-0.8	-2.7	-1.9	-4.6	-0.7	14.9	8.5	21.0	12.2	10.7
-9.4	1.9	-5.1	0.3	-5.2	-18.3	7.5	16.6	2.1	11.8	5.3
-10.6	-8.9	-9.9	-19.8	-9.4	-20.4		6.5	5.2		

**Table 2:** WAME weights for the COPD dataset. Weights in percent from estimate of differential expression using WAME on the COPD dataset.

No/mild emphysema				Severe emphysema					
	
-18.0	-6.7	-3.9	-8.9	11.8	2.6	12.0	4.0	12.6	7.6
-10.6	-7.3	-8.0	-5.6	7.1	9.0	6.7	0.9	6.2	5.5
-8.3	-3.6	-14.9	-4.3	-0.3	1.6	3.2	7.6	4.3	-2.5

**Figure 3:**
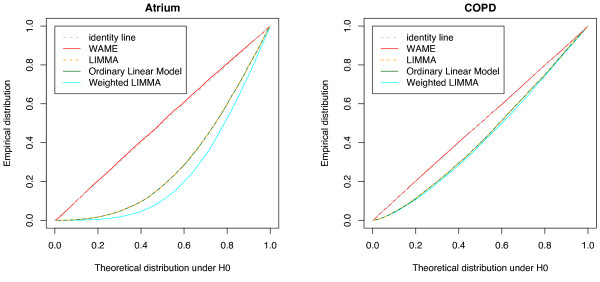
**Observed probability plots**. Empirical distribution of p-values compared to the distribution expected for non-differentially expressed genes. The OLM and LIMMA curves largely coincide, as does the identity line and the WAME curve.

The analysis methods described in Background are applied to the data and p-values and ranks computed. The respective probability plots are shown in Figure 3, demonstrating that there are substantial differences in the distribution of p-values between the different statistics. Since correlations and unequal variances are observed, the model assumptions of the alternative standard methods do not seem to hold. The p-values could thereby have become optimistic. On the other hand, it cannot be ruled out that the temporary assumption in WAME of no regulated genes makes its p-values conservative, which could also partly explain the differences. These problems are studied below by use of resampled data.

A common alternative to using the p-values as measures of significance is to consider the ranking of the genes, induced by the p-values or test statistics, and to select a fixed number of top ranked genes for further investigations. In Table 3 and Table 4 the concordance of the ranked lists are shown. The results from the included methods differ, for instance those from WAME compared to the other methods. This is not surprising since high correlations and highly unequal variances were identified by WAME, giving rise to highly unequal weights.

**Table 3:** Concordance of top lists in the Atrium dataset. Number of mutually included genes in the top-100 lists in the Atrium dataset as determined by the different methods.

	WAME	LIMMA	wLIMMA	OLM	FC
WAME	100	47	45	44	15
LIMMA	47	100	80	88	26
wLIMMA	45	80	100	76	21
OLM	44	88	76	100	21
FC	15	26	21	21	100

**Table 4:** Concordance of top lists in the COPD dataset. Number of mutually included genes in the top-100 lists in the COPD dataset as determined by the different methods.

	WAME	LIMMA	wLIMMA	OLM	FC
WAME	100	46	47	41	22
LIMMA	46	100	77	78	35
wLIMMA	47	77	100	66	32
OLM	41	78	66	100	25
FC	22	35	32	25	100

#### Resampled data

To examine closer the effect of violated assumptions of independence and identical distribution, we repeatedly selected two random subgroups of four arrays from within one group in the original data and performed tests between those groups. This was performed 100 times for the largest group in each of the two real datasets. Differentially expressed genes have unequal expected values in the two populations being sampled (cf. (2)). Since we now sample twice from the same condition, no differentially expressed genes exist.

**Figure 4:**
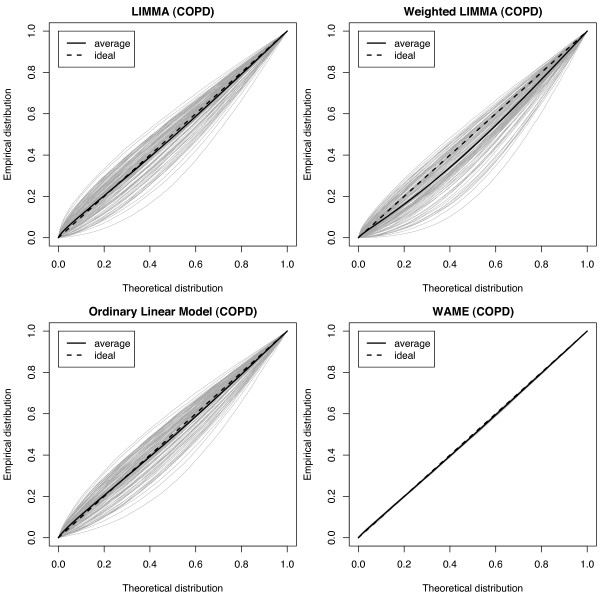
**Probability plots**. Empirical distributions of p-values for LIMMA, weighted LIMMA, OLM and WAME from tests on 100 resamples from the COPD dataset. Average empirical distribution indicated. Since no signal is added, the curves should ideally follow the diagonal.

Figure 4 shows the empirical p-value distributions for the resampled COPD data analysed with the four methods, together with the respective average empirical distribution,

F¯(p)=1100∑i=1100Fi(p),
 MathType@MTEF@5@5@+=feaafiart1ev1aaatCvAUfKttLearuWrP9MDH5MBPbIqV92AaeXatLxBI9gBaebbnrfifHhDYfgasaacH8akY=wiFfYdH8Gipec8Eeeu0xXdbba9frFj0=OqFfea0dXdd9vqai=hGuQ8kuc9pgc9s8qqaq=dirpe0xb9q8qiLsFr0=vr0=vr0dc8meaabaqaciaacaGaaeqabaqabeGadaaakeaacuWGgbGrgaqeaiabcIcaOiabdchaWjabcMcaPiabg2da9maalaaabaGaeGymaedabaGaeGymaeJaeGimaaJaeGimaadaamaaqahabaGaemOray0aaSbaaSqaaiabdMgaPbqabaGccqGGOaakcqWGWbaCcqGGPaqkaSqaaiabdMgaPjabg2da9iabigdaXaqaaiabigdaXiabicdaWiabicdaWaqdcqGHris5aOGaeiilaWcaaa@44D0@

where *F*_*i *_denotes the empirical CDF from the *i*th of the 100 resamples. For WAME, the p-value distributions are very close to the expected uniform. For OLM, LIMMA and weighted LIMMA there is a high variability between the p-value distributions and they are in many cases substantially different from the expected uniform. For WAME, OLM and LIMMA, the respective average empirical distribution is approximately correct, while for weighted LIMMA it is clearly optimistic. The results for the Atrium dataset (see Additional file 3) are very similar.

#### Evaluation of power

To evaluate the power of the tests in the studied datasets, a known regulation is added to randomly selected genes in one of the resampled groups, created according to the previous section. Thus, the noise is obtained from the real data and only the signal is synthetic. Ideally, the power can then be estimated by the proportion of differentially expressed genes that have a computed p-value less than a fixed level. However, valid p-values of the test statistics cannot be obtained from the respective models since, as demonstrated above, the corresponding assumptions are typically not valid. Ideally, the p-values would be determined by the true null distribution of the respective test statistics, given the array-wise quality deviations. In the simulation study, the critical value of the test statistics are therefore estimated from the empirical distribution of the test statistic for the unregulated genes. This is used to estimate the power of the different statistics (details are given in Methods).

**Figure 5:**
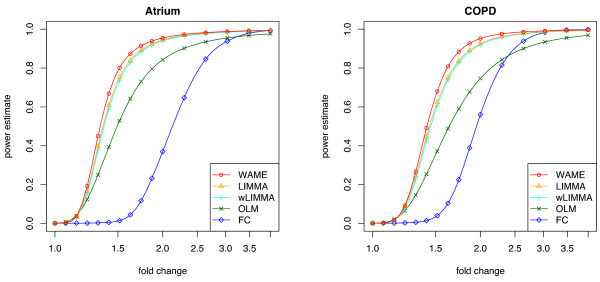
**Estimated power**. Estimated power in the simulated data for level 0.1% tests, based on resamples from the respective larger group in the Atrium and COPD datasets. Power is estimated at the marked points and spline interpolation is used in between.

**Figure 6:**
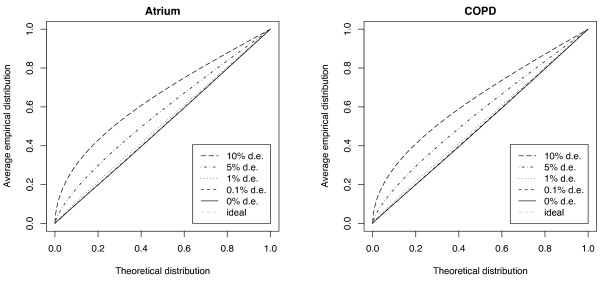
**Average empirical p-value distribution for WAME under regulation**. Average empirical p-value distribution of the unregulated genes, calculated using WAME, when 0%, 0.1%, 1%, 5% and 10% of the genes have a log_2 _differential expression of 1, i.e. a two-fold change. When genes are regulated the estimate of Σ is biased, leading to conservative, non-diagonal curves.

The power estimates for the different methods are shown in Figure 5, for a level 0.1% test. The 0.1% level yields approximately 22 false positives if relatively few genes are in fact differentially expressed. For WAME, Σ is estimated both before and after adding a signal to 2228 genes (10%), thereby substantially affecting the estimate of Σ (cf. Figure 6). The powers of the two versions are nevertheless very similar (difference less than 0.003) and only the latter version is included in the plot.

When the covariance-structure matrix Σ is estimated in WAME it is assumed that no genes are differentially expressed. Figure 6 includes the average empirical distribution for the p-values of the unregulated genes when different proportions of the genes have a log_2 _differential expression of 1. It is clear that the distributions are biased for high proportions, giving conservative p-values, which should be an effect of biased estimates of Σ.

The results from the studied datasets indicate (i) that WAME offers a relevant power increase compared to the included alternatives, (ii) that weighted LIMMA does not offer an advantage compared to LIMMA and (iii) that the moderated statistics (WAME, LIMMA and wLIMMA) are superior to the traditional methods of ranking by ordinary t-statistic (OLM) or estimated differential expression (FC).

## Discussion

### The WAME model and the simulations

The WAME model aims at catching quality deviations by one covariance-structure matrix common for all genes. This is certainly simplistic in some cases, e.g. when only certain physical parts of an array or certain types of mRNAs are of decreased quality. The estimated covariance structure can then only be expected to reflect a mixture of the qualities of the different genes. However, examining the simulations (Figure 5), we see a clear power gain in the WAME model compared to the other models. Also, WAME succeeds in catching enough of the quality deviations to make the p-value distributions more correct, thus providing increased usefulness of the p-values (Figure 3).

The models of LIMMA, weighted LIMMA and WAME are nested, where weighted LIMMA adds unequal variances and WAME adds unequal variances and correlations. Examination of Figure 1 shows that there are evident differences in variability between arrays. It is therefore interesting that we have not found a power increase of weighted LIMMA compared to LIMMA. Further, the p-values of weighted LIMMA turned out to be too optimistic (Figure 4). Comparison with the results of the WAME method, where the power increases and the p-value distributions get substantially more correct, suggests that the correlations are crucial in the model.

In the simulations, noise is taken from real data through resampling within a fixed group. This procedure provides data with fewer assumption on the noise structure compared to a fully parameterised simulation and should hopefully better reflect realistic situations. To evaluate the power of the different methods, a synthetic signal which is constant within each condition is added to the resample-based noise. This follows the assumption in the models of both WAME, OLM, LIMMA and weighted LIMMA, that the noise structure is equal for genes that are differentially expressed and non-differentially expressed. However, the biological variability of the expression of differentially expressed genes might be different under the different conditions due to the changed rôle of those genes. For complicated conditions such as complex diseases, the problem is more severe (cf. [15-17]) since crucial genes might only be differentially expressed in a subset of the studied arrays. Further work is needed to evaluate the performance of WAME in such settings, as well as to possibly expand it to better fit these situations.

A relevant question regarding the modelling of quality deviations by the covariance-structure matrix Σ is whether biologically interesting features may be hidden by this model. In the present datasets, the question can partly be answered by examining the pairwise plots (cf. Figure 2) and noticing that a large proportion of the genes show similar deviations, which should speak against a specific interesting biological explanation. Also, the estimated covariance structure matrix Σ can be inspected with the goal of finding relevant correlations between arrays and thus highlighting interesting features in the data. Possible future work is to use such inspections to reveal unwanted features in normalisation or in preprocessing wet-lab steps that give rise to correlated errors for a large proportion of the genes.

### Weights with switched signs

In the studied datasets, strong correlations combined with unequal variances make some weights within a group switch sign, in essence meaning that it is beneficial to partly subtract some arrays within a group in the estimate to be able to add more of the others in the same group (cf. Table 1 and Table 2). Since this might seem counter-intuitive, an elucidating example of possible mechanisms behind such weights follows.

Consider an example where two two-colour arrays are observed, *X*_1 _and *X*_2_. Let the two arrays have two sources of variation, one that is mutually independent (*ε*_1_, *ε*_2_) and one consisting of different proportions, *a*_1 _and *a*_2_, of one common source of variation *η*. Let *ε*_1_, *ε*_2 _and *η *be independent and normally distributed with expectation 0 and variances σε2
 MathType@MTEF@5@5@+=feaafiart1ev1aaatCvAUfKttLearuWrP9MDH5MBPbIqV92AaeXatLxBI9gBaebbnrfifHhDYfgasaacH8akY=wiFfYdH8Gipec8Eeeu0xXdbba9frFj0=OqFfea0dXdd9vqai=hGuQ8kuc9pgc9s8qqaq=dirpe0xb9q8qiLsFr0=vr0=vr0dc8meaabaqaciaacaGaaeqabaqabeGadaaakeaaiiGacqWFdpWCdaqhaaWcbaGae8xTdugabaGaeGOmaidaaaaa@3137@ and ση2
 MathType@MTEF@5@5@+=feaafiart1ev1aaatCvAUfKttLearuWrP9MDH5MBPbIqV92AaeXatLxBI9gBaebbnrfifHhDYfgasaacH8akY=wiFfYdH8Gipec8Eeeu0xXdbba9frFj0=OqFfea0dXdd9vqai=hGuQ8kuc9pgc9s8qqaq=dirpe0xb9q8qiLsFr0=vr0=vr0dc8meaabaqaciaacaGaaeqabaqabeGadaaakeaaiiGacqWFdpWCdaqhaaWcbaGae83TdGgabaGaeGOmaidaaaaa@313C@, respectively. Furthermore, let *μ *be the parameter to be estimated. The model becomes

*X*_*i *_= *μ *+ *a*_*i*_*η *+ *ε*_*i*_, *i *∈ {1,2}.

Then, *X*_1 _gets a negative weight if and only if

a1>a2+σε2a2ση2,
 MathType@MTEF@5@5@+=feaafiart1ev1aaatCvAUfKttLearuWrP9MDH5MBPbIqV92AaeXatLxBI9gBaebbnrfifHhDYfgasaacH8akY=wiFfYdH8Gipec8Eeeu0xXdbba9frFj0=OqFfea0dXdd9vqai=hGuQ8kuc9pgc9s8qqaq=dirpe0xb9q8qiLsFr0=vr0=vr0dc8meaabaqaciaacaGaaeqabaqabeGadaaakeaacqWGHbqydaWgaaWcbaGaeGymaedabeaakiabg6da+iabdggaHnaaBaaaleaacqaIYaGmaeqaaOGaey4kaSYaaSaaaeaaiiGacqWFdpWCdaqhaaWcbaGae8xTdugabaGaeGOmaidaaaGcbaGaemyyae2aaSbaaSqaaiabikdaYaqabaGccqWFdpWCdaqhaaWcbaGae83TdGgabaGaeGOmaidaaaaakiabcYcaSaaa@4000@

i.e. if array 1 includes a large enough contribution from the common source of variation. When a negative weight is allowed instead of removing the array, a smaller proportion of the common source of variation is included in the final estimate. Its precision is thus increased.

### Validity of the p-values and derived entities

Varying quality of arrays and correlated errors were demonstrated in [2,3] and in the present paper through examination of the data. These questions are typically neglected in microarray analyses, both when using parametric and when using non-parametric analysis methods, since independence and identical distribution or exchangeability are generally assumed under the null hypothesis. Thus, the validity is questionable of the corresponding p-values and their derived entities, e.g. false discovery rates and estimates of proportions of differentially expressed genes. This problem is obvious in the resample based simulations.

A number of experiments have been analysed (data not shown) in addition to those published in the present paper and in [2,3]. In almost all cases relevant unequal variances and correlations have been identified, indicating that the problem is common.

In the resample based simulations with added signal, WAME is shown to be conservative, which is an effect of the biased estimate of Σ. Further work on an estimator of Σ with better characteristics under regulation is therefore needed. However, the simulations indicate (i) that the power of the test is basically unaffected by the bias and (ii) that hundreds of genes may be differentially expressed (two-fold) with only mildly conservative p-values as result.

### Correlations between genes or between arrays?

It has recently been argued that the expression of different genes are highly dependent, making the law of large number normally inapplicable [18] and standard estimators of e.g. the false discovery rate (FDR) imprecise [19]. In [19], a latent FDR is introduced, being the conditional FDR given a random effect *b *that captures the correlation effects between genes. The FDR is then the marginal latent FDR, that is the average over the random effect *b*.

For the datasets examined in the present paper, the model assumptions of e.g. the ordinary linear model are shown not to hold (cf. Figure 1 and Figure 2). This can be expected to result in invalid p-values, which is indeed observed in Figure 4. Interestingly, the p-value distribution seem to be valid marginally, i.e. on average over the different resamples, which would yield valid but imprecise estimates of the FDR. This type of failed model assumptions is not taken into account in e.g. [18,19]. Since for a performed experiment, the p-values from the ordinary t-statistic (OLM) share a common bias conditional on the experiment (see Figure 4), the different p-values may be highly dependent. However, this dependency is due to failure of taking array-wide quality deviations into account in the model and not due to the nature of microarray data *per se*, e.g. through substantial long-range gene-gene interactions.

Consequently, the strong observed dependencies between statistics from different genes might largely be explainable by quality deviations between the arrays in the experiment, e.g. correlations between arrays. Since WAME models these deviations such that the p-values are essentially correctly distributed when few genes are differentially expressed in the studied datasets, the dependency between genes should be greatly decreased. The covariance structure matrix Σ is therefore in a sense a parallel to the random factor *b *in [19]. It remains as future work to evaluate the gene-gene dependencies and estimates of e.g. the FDR in the context of the WAME model.

In the WAME model, the data from different genes are assumed independent, which is unrealistic, e.g. since genes act together in pathways. However, this is only used in the derivation of the maximum likelihood estimaties of the covariance structure matrix Σ and the shape parameter *α*. The assumption could thus be relaxed to a dependence between the different genes that is weak enough that the estimates of Σ and *α *become precise, and accurate under *H*_0_. This holds if the law of large numbers is applicable for averages of certain functions of the gene-wise observed data (cf. the likelihood functions in [2,3]). Given the large number of genes and the observed p-value distributions in Figure 4, this relaxed assumption seems plausible.

It can be noted that for the studied data, WAME has higher power and considerably more valid p-values than weighted LIMMA. Since the difference between the weighted LIMMA and WAME models is the inclusion of correlations between arrays, this emphasises the importance of the correlations in the model.

## Conclusion

Statistical methods in microarray analysis are typically based on the often erroneous assumption that the data from different arrays are independent and identically distributed. An exception is Weighted Analysis of Microarray Experiment (WAME) where heteroscedasticity and correlations between arrays are modelled by a covariance-structure common for all genes. In the present paper, WAME has been extended to handle datasets without a natural pairing, e.g. data from one-channel microarrays, and corresponding estimates and test statistics have been derived. In the examined one-channel microarray datasets WAME detected unequal variances and nonzero correlations.

WAME was compared with four other common methods: an ordinary linear model with t-tests, LIMMA, weighted LIMMA, and fold-change ranking. The comparison was performed using resampling of the different arrays within the datasets. Here, WAME had the highest power. When a relatively small proportion of the genes are regulated, WAME also generates close to correct p-value distributions while the p-value distributions from the other methods are highly variable. However, when many genes are differentially expressed, the p-values from WAME tend to be conservative.

In conclusion, p-values from the standard methods for microarray analysis should in general not be trusted and any result based on p-values, e.g. estimates of the number of regulated genes and false discovery rates, should be interpreted with care. The analyses of the examined datasets showed that WAME gives a powerful approach for finding differentially expressed genes and that it produces more trustworthy p-values when a relatively small proportion of genes are differentially expressed.

## Methods

### Details on the new version of WAME

#### Model Framework

For *g *= 1,..., *m*, let **X**_***g ***_be an *n*-dimensional vector with expectation ***μ***_***g ***_= *D**γ***_***g***_, where *D *is the design matrix, having rank *k*, and ***γ***_***g ***_∈ ℝ^*q *^is the parameter vector. Furthermore, let

**X**_***g ***_| *c*_*g *_~ N(***μ***_***g***_, *c*_*g*_Σ),

*c*_*g *_~ Γ^-1^(*α*, 1),

where Σ is the non-singular covariance-structure matrix, *c*_*g *_is the variance-scaling factor, *α *is the shape parameter for *c*_*g *_and (*c*_1_, **X**_1_),...,(*c*_*m*_, **X**_*m*_) are assumed independent. The differential expression vector is defined as

***δ***_***g ***_= *C**γ***_***g***_,

where *C *is a matrix of rank *p *such that ***δ***_***g ***_is estimable. Here, an estimator of ***δ***_***g ***_and a test for

H0:δg=0HA:δg≠0
 MathType@MTEF@5@5@+=feaafiart1ev1aaatCvAUfKttLearuWrP9MDH5MBPbIqV92AaeXatLxBI9gBaebbnrfifHhDYfgasaacH8akY=wiFfYdH8Gipec8Eeeu0xXdbba9frFj0=OqFfea0dXdd9vqai=hGuQ8kuc9pgc9s8qqaq=dirpe0xb9q8qiLsFr0=vr0=vr0dc8meaabaqaciaacaGaaeqabaqabeGadaaakeaafaqadeGadaaabaGaemisaG0aaSbaaSqaaiabicdaWaqabaGccqGG6aGoiiWacqWF0oazdaWgaaWcbaGaem4zaCgabeaaaOqaaiabg2da9aqaaGqabiab+bdaWaqaaiabdIeainaaBaaaleaacqWGbbqqaeqaaOGaeiOoaOJae8hTdq2aaSbaaSqaaiabdEgaNbqabaaakeaacqGHGjsUaeaacqGFWaamaaaaaa@3E56@

are in focus.

As mentioned in Background, one main obstacle is that Σ is hard to estimate. In fact, Σ and ***δ***_***g ***_cannot be maximum likelihood estimated simultaneously, since there are trivial infinite suprema of the likelihood, e.g. when the variance of one observation is set to zero and the corresponding mean is selected so that it equals that observation.

#### The current WAME method

In the current version of WAME [3], Σ is estimated as follows. First, temporarily assume that ***μ***_***g ***_= **0 **for all genes, which is reasonable for paired experimental designs with few differentially expressed genes between any pairwise measured conditions. For each gene, the variance scaling factor *c*_*g *_is removed by dividing the *n *measurements with the first measurement, yielding a random vector distributed according to a multivariate generalisation of the Cauchy distribution. A scaled version of Σ is then maximum likelihood estimated numerically. Second, the unknown scale and the hyperparameter *α *of the prior distribution of *c*_*g *_are maximum likelihood estimated numerically without the assumption of ***μ***_***g ***_= **0**. The parameters Σ and *α *are subsequently treated as known in the maximum-likelihood estimates and likelihood-ratio tests for the different genes.

#### The new WAME method

The new version of WAME relaxes the assumption from ***μ***_***g ***_= **0 **to ***δ***_***g ***_= **0**, which incorporates many designs without a natural pairing. This is performed by subtracting an arbitrary estimator μ˜g0
 MathType@MTEF@5@5@+=feaafiart1ev1aaatCvAUfKttLearuWrP9MDH5MBPbIqV92AaeXatLxBI9gBaebbnrfifHhDYfgasaacH8akY=wiFfYdH8Gipec8Eeeu0xXdbba9frFj0=OqFfea0dXdd9vqai=hGuQ8kuc9pgc9s8qqaq=dirpe0xb9q8qiLsFr0=vr0=vr0dc8meaabaqaciaacaGaaeqabaqabeGadaaakeaaiiWacuWF8oqBgaacamaaDaaaleaacqWGNbWzaeaacqaIWaamaaaaaa@30EB@ of ***μ***_***g***_, which is unbiased under *H*_0 _and has as image the space V
 MathType@MTEF@5@5@+=feaafiart1ev1aaatCvAUfKttLearuWrP9MDH5MBPbIqV92AaeXatLxBI9gBaebbnrfifHhDYfgasaacH8akY=wiFfYdH8Gipec8Eeeu0xXdbba9frFj0=OqFfea0dXdd9vqai=hGuQ8kuc9pgc9s8qqaq=dirpe0xb9q8qiLsFr0=vr0=vr0dc8meaabaqaciaacaGaaeqabaqabeGadaaakeaat0uy0HwzTfgDPnwy1egaryqtHrhAL1wy0L2yHvdaiqaacqWFveVvaaa@384A@_0 _of possible values for ***μ***_***g ***_under *H*_0_,

Yg=Xg−μ˜g0.
 MathType@MTEF@5@5@+=feaafiart1ev1aaatCvAUfKttLearuWrP9MDH5MBPbIqV92AaeXatLxBI9gBaebbnrfifHhDYfgasaacH8akY=wiFfYdH8Gipec8Eeeu0xXdbba9frFj0=OqFfea0dXdd9vqai=hGuQ8kuc9pgc9s8qqaq=dirpe0xb9q8qiLsFr0=vr0=vr0dc8meaabaqaciaacaGaaeqabaqabeGadaaakeaaieqacqWFzbqwdaWgaaWcbaGaem4zaCgabeaakiabg2da9iab=HfaynaaBaaaleaacqWGNbWzaeqaaOGaeyOeI0cccmGaf4hVd0MbaGaadaqhaaWcbaGaem4zaCgabaGaeGimaadaaOGaeiOla4caaa@395B@

It can be shown that this transformation preserves the estimability of ***δ***_***g***_.

By construction, the transformed data **Y**_***g ***_will have expectation zero for non-differentially expressed genes and the current WAME method can be applied on **Y**_***g***_, including the estimation of the covariance-structure matrix Σ_*Y *_for **Y**_***g***_. It will now be proved that the likelihood ratio tests of (9) and the maximum likelihood estimates of ***δ***_***g ***_based on **X**_***g ***_or **Y**_***g ***_are identical, if *α *and Σ or Σ_*Y *_respectively are considered known. 

We shall henceforth consider a fixed gene *g *and drop the *g *index.

#### Equality of tests and estimators

Before beginning, some further definitions are needed. Define the Mahalanobis inner product corresponding to a symmetric *n *by *n *matrix *A *as

〈x1,x2〉A=x1TA−x2,
 MathType@MTEF@5@5@+=feaafiart1ev1aaatCvAUfKttLearuWrP9MDH5MBPbIqV92AaeXatLxBI9gBaebbnrfifHhDYfgasaacH8akY=wiFfYdH8Gipec8Eeeu0xXdbba9frFj0=OqFfea0dXdd9vqai=hGuQ8kuc9pgc9s8qqaq=dirpe0xb9q8qiLsFr0=vr0=vr0dc8meaabaqaciaacaGaaeqabaqabeGadaaakeaacqGHPms4ieqacqWF4baEdaWgaaWcbaGaeGymaedabeaakiabcYcaSiab=Hha4naaBaaaleaacqaIYaGmaeqaaOGaeyOkJe=aaSbaaSqaaiabdgeabbqabaGccqGH9aqpcqWF4baEdaqhaaWcbaGaeGymaedabaGaeeivaqfaaOGaemyqae0aaWbaaSqabeaacqGHsislaaGccqWF4baEdaWgaaWcbaGaeGOmaidabeaakiabcYcaSaaa@420F@

and the norm ∥·∥_*A *_as

‖x‖A2=〈x,x〉=xTA−x,
 MathType@MTEF@5@5@+=feaafiart1ev1aaatCvAUfKttLearuWrP9MDH5MBPbIqV92AaeXatLxBI9gBaebbnrfifHhDYfgasaacH8akY=wiFfYdH8Gipec8Eeeu0xXdbba9frFj0=OqFfea0dXdd9vqai=hGuQ8kuc9pgc9s8qqaq=dirpe0xb9q8qiLsFr0=vr0=vr0dc8meaabaqaciaacaGaaeqabaqabeGadaaakeaadaqbdaqaaGqabiab=Hha4bGaayzcSlaawQa7amaaDaaaleaacqWGbbqqaeaacqaIYaGmaaGccqGH9aqpcqGHPms4cqWF4baEcqGGSaalcqWF4baEcqGHQms8cqGH9aqpcqWF4baEdaahaaWcbeqaaiabbsfaubaakiabdgeabnaaCaaaleqabaGaeyOeI0caaOGae8hEaGNaeiilaWcaaa@443E@

where **x**, **x**_1_, **x**_2 _lies in the rowspace of *A *and the generalised inverse *A*^^-^^is any matrix satisfying *AA*^-^*A *= *A*. Let *χ *denote the *n*-dimensional inner product space with ⟨·,·⟩_Σ _as inner product. Define V
 MathType@MTEF@5@5@+=feaafiart1ev1aaatCvAUfKttLearuWrP9MDH5MBPbIqV92AaeXatLxBI9gBaebbnrfifHhDYfgasaacH8akY=wiFfYdH8Gipec8Eeeu0xXdbba9frFj0=OqFfea0dXdd9vqai=hGuQ8kuc9pgc9s8qqaq=dirpe0xb9q8qiLsFr0=vr0=vr0dc8meaabaqaciaacaGaaeqabaqabeGadaaakeaat0uy0HwzTfgDPnwy1egaryqtHrhAL1wy0L2yHvdaiqaacqWFveVvaaa@384A@ ⊂ *χ *as the space of possible values for ***μ***_***g***_,

V
 MathType@MTEF@5@5@+=feaafiart1ev1aaatCvAUfKttLearuWrP9MDH5MBPbIqV92AaeXatLxBI9gBaebbnrfifHhDYfgasaacH8akY=wiFfYdH8Gipec8Eeeu0xXdbba9frFj0=OqFfea0dXdd9vqai=hGuQ8kuc9pgc9s8qqaq=dirpe0xb9q8qiLsFr0=vr0=vr0dc8meaabaqaciaacaGaaeqabaqabeGadaaakeaat0uy0HwzTfgDPnwy1egaryqtHrhAL1wy0L2yHvdaiqaacqWFveVvaaa@384A@ = {***μ ***: ***μ ***= *D**γ***, ***γ ***∈ ℝ^*q*^}

and let V
 MathType@MTEF@5@5@+=feaafiart1ev1aaatCvAUfKttLearuWrP9MDH5MBPbIqV92AaeXatLxBI9gBaebbnrfifHhDYfgasaacH8akY=wiFfYdH8Gipec8Eeeu0xXdbba9frFj0=OqFfea0dXdd9vqai=hGuQ8kuc9pgc9s8qqaq=dirpe0xb9q8qiLsFr0=vr0=vr0dc8meaabaqaciaacaGaaeqabaqabeGadaaakeaat0uy0HwzTfgDPnwy1egaryqtHrhAL1wy0L2yHvdaiqaacqWFveVvaaa@384A@_0 _⊂ *χ *denote the corresponding space restricted by the null hypothesis,

V
 MathType@MTEF@5@5@+=feaafiart1ev1aaatCvAUfKttLearuWrP9MDH5MBPbIqV92AaeXatLxBI9gBaebbnrfifHhDYfgasaacH8akY=wiFfYdH8Gipec8Eeeu0xXdbba9frFj0=OqFfea0dXdd9vqai=hGuQ8kuc9pgc9s8qqaq=dirpe0xb9q8qiLsFr0=vr0=vr0dc8meaabaqaciaacaGaaeqabaqabeGadaaakeaat0uy0HwzTfgDPnwy1egaryqtHrhAL1wy0L2yHvdaiqaacqWFveVvaaa@384A@_0 _= {***μ ***: ***μ ***= *D**γ***, *C**γ ***= **0**, ***γ ***∈ ℝ^*q*^}.

**Proposition ***Let *μ˜0
 MathType@MTEF@5@5@+=feaafiart1ev1aaatCvAUfKttLearuWrP9MDH5MBPbIqV92AaeXatLxBI9gBaebbnrfifHhDYfgasaacH8akY=wiFfYdH8Gipec8Eeeu0xXdbba9frFj0=OqFfea0dXdd9vqai=hGuQ8kuc9pgc9s8qqaq=dirpe0xb9q8qiLsFr0=vr0=vr0dc8meaabaqaciaacaGaaeqabaqabeGadaaakeaaiiWacuWF8oqBgaacamaaCaaaleqabaGaeGimaadaaaaa@2F94@*be an arbitrary linear estimator of ****μ***, *which is unbiased under H*_0 _*and which has image *V
 MathType@MTEF@5@5@+=feaafiart1ev1aaatCvAUfKttLearuWrP9MDH5MBPbIqV92AaeXatLxBI9gBaebbnrfifHhDYfgasaacH8akY=wiFfYdH8Gipec8Eeeu0xXdbba9frFj0=OqFfea0dXdd9vqai=hGuQ8kuc9pgc9s8qqaq=dirpe0xb9q8qiLsFr0=vr0=vr0dc8meaabaqaciaacaGaaeqabaqabeGadaaakeaat0uy0HwzTfgDPnwy1egaryqtHrhAL1wy0L2yHvdaiqaacqWFveVvaaa@384A@_0_. *Let*

Y=X−μ˜0,
 MathType@MTEF@5@5@+=feaafiart1ev1aaatCvAUfKttLearuWrP9MDH5MBPbIqV92AaeXatLxBI9gBaebbnrfifHhDYfgasaacH8akY=wiFfYdH8Gipec8Eeeu0xXdbba9frFj0=OqFfea0dXdd9vqai=hGuQ8kuc9pgc9s8qqaq=dirpe0xb9q8qiLsFr0=vr0=vr0dc8meaabaqaciaacaGaaeqabaqabeGadaaakeaaieqacqWFzbqwcqGH9aqpcqWFybawcqGHsisliiWacuGF8oqBgaacamaaCaaaleqabaGaeGimaadaaOGaeiilaWcaaa@34E6@

*and let *Σ_*Y *_*be the covariance-structure matrix of ***Y**. *Then the likelihood ratio test of (9) and the maximum likelihood estimate of ****δ ****based on ***X ***with *Σ *and α known are identical to the ones based on ***Y ***with *Σ_*Y *_*and α known*.

### Proof of the Proposition

The proof is divided into two steps which combined conclude the proof.

1. The likelihood ratio test (LRT) of (9) and the maximum likelihood estimator (MLE) of ***δ ***does not depend on the choice of μ˜0
 MathType@MTEF@5@5@+=feaafiart1ev1aaatCvAUfKttLearuWrP9MDH5MBPbIqV92AaeXatLxBI9gBaebbnrfifHhDYfgasaacH8akY=wiFfYdH8Gipec8Eeeu0xXdbba9frFj0=OqFfea0dXdd9vqai=hGuQ8kuc9pgc9s8qqaq=dirpe0xb9q8qiLsFr0=vr0=vr0dc8meaabaqaciaacaGaaeqabaqabeGadaaakeaaiiWacuWF8oqBgaacamaaCaaaleqabaGaeGimaadaaaaa@2F94@.

2. The proposition holds when μ˜0
 MathType@MTEF@5@5@+=feaafiart1ev1aaatCvAUfKttLearuWrP9MDH5MBPbIqV92AaeXatLxBI9gBaebbnrfifHhDYfgasaacH8akY=wiFfYdH8Gipec8Eeeu0xXdbba9frFj0=OqFfea0dXdd9vqai=hGuQ8kuc9pgc9s8qqaq=dirpe0xb9q8qiLsFr0=vr0=vr0dc8meaabaqaciaacaGaaeqabaqabeGadaaakeaaiiWacuWF8oqBgaacamaaCaaaleqabaGaeGimaadaaaaa@2F94@ is the MLE of ***μ ***under *H*_0_.

#### Proof of step 1

Let ***μ' ***and ***μ'' ***be two valid choices of μ˜0
 MathType@MTEF@5@5@+=feaafiart1ev1aaatCvAUfKttLearuWrP9MDH5MBPbIqV92AaeXatLxBI9gBaebbnrfifHhDYfgasaacH8akY=wiFfYdH8Gipec8Eeeu0xXdbba9frFj0=OqFfea0dXdd9vqai=hGuQ8kuc9pgc9s8qqaq=dirpe0xb9q8qiLsFr0=vr0=vr0dc8meaabaqaciaacaGaaeqabaqabeGadaaakeaaiiWacuWF8oqBgaacamaaCaaaleqabaGaeGimaadaaaaa@2F94@, i.e. they are both unbiased estimators of ***μ ***under *H*_0 _and have V
 MathType@MTEF@5@5@+=feaafiart1ev1aaatCvAUfKttLearuWrP9MDH5MBPbIqV92AaeXatLxBI9gBaebbnrfifHhDYfgasaacH8akY=wiFfYdH8Gipec8Eeeu0xXdbba9frFj0=OqFfea0dXdd9vqai=hGuQ8kuc9pgc9s8qqaq=dirpe0xb9q8qiLsFr0=vr0=vr0dc8meaabaqaciaacaGaaeqabaqabeGadaaakeaat0uy0HwzTfgDPnwy1egaryqtHrhAL1wy0L2yHvdaiqaacqWFveVvaaa@384A@_0 _as image. Let **Y' **= **X **- ***μ' ***and **Y'' **= **X **- ***μ''***. Recall that a matrix *P *is a projection matrix projecting on V
 MathType@MTEF@5@5@+=feaafiart1ev1aaatCvAUfKttLearuWrP9MDH5MBPbIqV92AaeXatLxBI9gBaebbnrfifHhDYfgasaacH8akY=wiFfYdH8Gipec8Eeeu0xXdbba9frFj0=OqFfea0dXdd9vqai=hGuQ8kuc9pgc9s8qqaq=dirpe0xb9q8qiLsFr0=vr0=vr0dc8meaabaqaciaacaGaaeqabaqabeGadaaakeaat0uy0HwzTfgDPnwy1egaryqtHrhAL1wy0L2yHvdaiqaacqWFveVvaaa@384A@_0 _if and only if for all **x **∈ ℝ^*n*^, *Px *∈ V
 MathType@MTEF@5@5@+=feaafiart1ev1aaatCvAUfKttLearuWrP9MDH5MBPbIqV92AaeXatLxBI9gBaebbnrfifHhDYfgasaacH8akY=wiFfYdH8Gipec8Eeeu0xXdbba9frFj0=OqFfea0dXdd9vqai=hGuQ8kuc9pgc9s8qqaq=dirpe0xb9q8qiLsFr0=vr0=vr0dc8meaabaqaciaacaGaaeqabaqabeGadaaakeaat0uy0HwzTfgDPnwy1egaryqtHrhAL1wy0L2yHvdaiqaacqWFveVvaaa@384A@_0 _and for all **x**_0 _∈ V
 MathType@MTEF@5@5@+=feaafiart1ev1aaatCvAUfKttLearuWrP9MDH5MBPbIqV92AaeXatLxBI9gBaebbnrfifHhDYfgasaacH8akY=wiFfYdH8Gipec8Eeeu0xXdbba9frFj0=OqFfea0dXdd9vqai=hGuQ8kuc9pgc9s8qqaq=dirpe0xb9q8qiLsFr0=vr0=vr0dc8meaabaqaciaacaGaaeqabaqabeGadaaakeaat0uy0HwzTfgDPnwy1egaryqtHrhAL1wy0L2yHvdaiqaacqWFveVvaaa@384A@_0_, *P***x**_0 _= **x**_0_. It can be shown that ***μ' ***and ***μ'' ***can be written as ***μ' ***= *P' X *and ***μ'' ***= *P'' X *for some projection matrices *P' *and *P'' *projecting on V
 MathType@MTEF@5@5@+=feaafiart1ev1aaatCvAUfKttLearuWrP9MDH5MBPbIqV92AaeXatLxBI9gBaebbnrfifHhDYfgasaacH8akY=wiFfYdH8Gipec8Eeeu0xXdbba9frFj0=OqFfea0dXdd9vqai=hGuQ8kuc9pgc9s8qqaq=dirpe0xb9q8qiLsFr0=vr0=vr0dc8meaabaqaciaacaGaaeqabaqabeGadaaakeaat0uy0HwzTfgDPnwy1egaryqtHrhAL1wy0L2yHvdaiqaacqWFveVvaaa@384A@_0_. Since *P' *and *P'' *project on the same space it follows that *P' P'' *= *P'' *and *P'' P' *= *P'*, and thus (*I *- *P'*) **Y**'' = **Y**' and (*I *- *P''*) **Y**' = **Y**''. Hence there is an invertible map between **Y**' and **Y**'' and likelihood methods based on **Y**' and **Y**'' respectively will give equal results. Consequently, the MLE of (9) and the LRT of ***δ ***will not depend on the choice of μ˜0
 MathType@MTEF@5@5@+=feaafiart1ev1aaatCvAUfKttLearuWrP9MDH5MBPbIqV92AaeXatLxBI9gBaebbnrfifHhDYfgasaacH8akY=wiFfYdH8Gipec8Eeeu0xXdbba9frFj0=OqFfea0dXdd9vqai=hGuQ8kuc9pgc9s8qqaq=dirpe0xb9q8qiLsFr0=vr0=vr0dc8meaabaqaciaacaGaaeqabaqabeGadaaakeaaiiWacuWF8oqBgaacamaaCaaaleqabaGaeGimaadaaaaa@2F94@

#### Proof of step 2

Since ***δ ***is estimable based on **X**, there exist a matrix *A *such that *C *= *AD *and thus ***δ ***= *A****μ***. The likelihood of ***μ ***can therefore be examined instead of the likelihood of ***δ***.

The likelihood of ***μ ***based on **X **can be shown to be

L(μ|X)=∫0∞f(X|μ,c)⋅f(c)dc∝[‖X−μ‖Σ2/2+1]−n/2−α,
 MathType@MTEF@5@5@+=feaafiart1ev1aaatCvAUfKttLearuWrP9MDH5MBPbIqV92AaeXatLxBI9gBaebbnrfifHhDYfgasaacH8akY=wiFfYdH8Gipec8Eeeu0xXdbba9frFj0=OqFfea0dXdd9vqai=hGuQ8kuc9pgc9s8qqaq=dirpe0xb9q8qiLsFr0=vr0=vr0dc8meaabaqaciaacaGaaeqabaqabeGadaaakeaafaqaaeGadaaabaGaemitaWKaeiikaGcccmGae8hVd0MaeiiFaWhcbeGae4hwaGLaeiykaKcabaGaeyypa0dabaWaa8qmaeaacqWGMbGzcqGGOaakcqGFybawcqGG8baFcqWF8oqBcqGGSaalcqWGJbWycqGGPaqkcqGHflY1cqWGMbGzcqGGOaakcqWGJbWycqGGPaqkcqWGKbazcqWGJbWyaSqaaiabicdaWaqaaiabg6HiLcqdcqGHRiI8aaGcbaaabaGaeyyhIulabaWaamWaaeaadaqbdaqaaiab+HfayjabgkHiTiab=X7aTbGaayzcSlaawQa7amaaDaaaleaacqqHJoWuaeaacqaIYaGmaaGccqGGVaWlcqaIYaGmcqGHRaWkcqaIXaqmaiaawUfacaGLDbaadaahaaWcbeqaaiabgkHiTiabd6gaUjabc+caViabikdaYiabgkHiTGGaciab9f7aHbaakiabcYcaSaaaaaa@6514@

where ∝ denotes proportionality. Using the Projection Theorem [20], the MLE of ***μ ***is the orthogonal projection of **X **on V
 MathType@MTEF@5@5@+=feaafiart1ev1aaatCvAUfKttLearuWrP9MDH5MBPbIqV92AaeXatLxBI9gBaebbnrfifHhDYfgasaacH8akY=wiFfYdH8Gipec8Eeeu0xXdbba9frFj0=OqFfea0dXdd9vqai=hGuQ8kuc9pgc9s8qqaq=dirpe0xb9q8qiLsFr0=vr0=vr0dc8meaabaqaciaacaGaaeqabaqabeGadaaakeaat0uy0HwzTfgDPnwy1egaryqtHrhAL1wy0L2yHvdaiqaacqWFveVvaaa@384A@,

μ^=PVX,
 MathType@MTEF@5@5@+=feaafiart1ev1aaatCvAUfKttLearuWrP9MDH5MBPbIqV92AaeXatLxBI9gBaebbnrfifHhDYfgasaacH8akY=wiFfYdH8Gipec8Eeeu0xXdbba9frFj0=OqFfea0dXdd9vqai=hGuQ8kuc9pgc9s8qqaq=dirpe0xb9q8qiLsFr0=vr0=vr0dc8meaabaqaciaacaGaaeqabaqabeGadaaakeaaiiWacuWF8oqBgaqcaiabg2da9iabbcfaqnaaBaaaleaat0uy0HwzTfgDPnwy1egaryqtHrhAL1wy0L2yHvdaiqaacqGFveVvaeqaaGqabOGae0hwaGLaeiilaWcaaa@3E97@

where the orthogonality is according to the inner product of *χ*. When *H*_0 _is true, ***μ ***is restricted to V
 MathType@MTEF@5@5@+=feaafiart1ev1aaatCvAUfKttLearuWrP9MDH5MBPbIqV92AaeXatLxBI9gBaebbnrfifHhDYfgasaacH8akY=wiFfYdH8Gipec8Eeeu0xXdbba9frFj0=OqFfea0dXdd9vqai=hGuQ8kuc9pgc9s8qqaq=dirpe0xb9q8qiLsFr0=vr0=vr0dc8meaabaqaciaacaGaaeqabaqabeGadaaakeaat0uy0HwzTfgDPnwy1egaryqtHrhAL1wy0L2yHvdaiqaacqWFveVvaaa@384A@_0 _and thus the MLE of ***μ ***becomes

μ^0=PV0X.
 MathType@MTEF@5@5@+=feaafiart1ev1aaatCvAUfKttLearuWrP9MDH5MBPbIqV92AaeXatLxBI9gBaebbnrfifHhDYfgasaacH8akY=wiFfYdH8Gipec8Eeeu0xXdbba9frFj0=OqFfea0dXdd9vqai=hGuQ8kuc9pgc9s8qqaq=dirpe0xb9q8qiLsFr0=vr0=vr0dc8meaabaqaciaacaGaaeqabaqabeGadaaakeaaiiWacuWF8oqBgaqcamaaCaaaleqabaGaeGimaadaaOGaeyypa0Jaeeiuaa1aaSbaaSqaamrtHrhAL1wy0L2yHvtyaeHbnfgDOvwBHrxAJfwnaGabaiab+vr8wnaaBaaameaacqaIWaamaeqaaaWcbeaaieqakiab9HfayHqaaiab85caUaaa@40E9@

Note that μ^0
 MathType@MTEF@5@5@+=feaafiart1ev1aaatCvAUfKttLearuWrP9MDH5MBPbIqV92AaeXatLxBI9gBaebbnrfifHhDYfgasaacH8akY=wiFfYdH8Gipec8Eeeu0xXdbba9frFj0=OqFfea0dXdd9vqai=hGuQ8kuc9pgc9s8qqaq=dirpe0xb9q8qiLsFr0=vr0=vr0dc8meaabaqaciaacaGaaeqabaqabeGadaaakeaaiiWacuWF8oqBgaqcamaaCaaaleqabaGaeGimaadaaaaa@2F95@ is a valid choice for μ˜0
 MathType@MTEF@5@5@+=feaafiart1ev1aaatCvAUfKttLearuWrP9MDH5MBPbIqV92AaeXatLxBI9gBaebbnrfifHhDYfgasaacH8akY=wiFfYdH8Gipec8Eeeu0xXdbba9frFj0=OqFfea0dXdd9vqai=hGuQ8kuc9pgc9s8qqaq=dirpe0xb9q8qiLsFr0=vr0=vr0dc8meaabaqaciaacaGaaeqabaqabeGadaaakeaaiiWacuWF8oqBgaacamaaCaaaleqabaGaeGimaadaaaaa@2F94@, i.e. μ^0
 MathType@MTEF@5@5@+=feaafiart1ev1aaatCvAUfKttLearuWrP9MDH5MBPbIqV92AaeXatLxBI9gBaebbnrfifHhDYfgasaacH8akY=wiFfYdH8Gipec8Eeeu0xXdbba9frFj0=OqFfea0dXdd9vqai=hGuQ8kuc9pgc9s8qqaq=dirpe0xb9q8qiLsFr0=vr0=vr0dc8meaabaqaciaacaGaaeqabaqabeGadaaakeaaiiWacuWF8oqBgaqcamaaCaaaleqabaGaeGimaadaaaaa@2F95@ is unbiased under *H*_0 _and has V
 MathType@MTEF@5@5@+=feaafiart1ev1aaatCvAUfKttLearuWrP9MDH5MBPbIqV92AaeXatLxBI9gBaebbnrfifHhDYfgasaacH8akY=wiFfYdH8Gipec8Eeeu0xXdbba9frFj0=OqFfea0dXdd9vqai=hGuQ8kuc9pgc9s8qqaq=dirpe0xb9q8qiLsFr0=vr0=vr0dc8meaabaqaciaacaGaaeqabaqabeGadaaakeaat0uy0HwzTfgDPnwy1egaryqtHrhAL1wy0L2yHvdaiqaacqWFveVvaaa@384A@_0 _as image. Let

Z=X−μ^0
 MathType@MTEF@5@5@+=feaafiart1ev1aaatCvAUfKttLearuWrP9MDH5MBPbIqV92AaeXatLxBI9gBaebbnrfifHhDYfgasaacH8akY=wiFfYdH8Gipec8Eeeu0xXdbba9frFj0=OqFfea0dXdd9vqai=hGuQ8kuc9pgc9s8qqaq=dirpe0xb9q8qiLsFr0=vr0=vr0dc8meaabaqaciaacaGaaeqabaqabeGadaaakeaaieqacqWFAbGwcqGH9aqpcqWFybawcqGHsisliiWacuGF8oqBgaqcamaaCaaaleqabaGaeGimaadaaaaa@33FF@

which gives Z=PV0⊥X
 MathType@MTEF@5@5@+=feaafiart1ev1aaatCvAUfKttLearuWrP9MDH5MBPbIqV92AaeXatLxBI9gBaebbnrfifHhDYfgasaacH8akY=wiFfYdH8Gipec8Eeeu0xXdbba9frFj0=OqFfea0dXdd9vqai=hGuQ8kuc9pgc9s8qqaq=dirpe0xb9q8qiLsFr0=vr0=vr0dc8meaabaqaciaacaGaaeqabaqabeGadaaakeaaieqacqWFAbGwcqGH9aqpcqqGqbaudaWgaaWcbaWenfgDOvwBHrxAJfwnHbqeg0uy0HwzTfgDPnwy1aaceaGae4xfXB1aa0baaWqaaiabicdaWaqaambvLH1qnnvETj2BSbqehmwBZLxmWaacfaGae0xPI8daaaWcbeaakiab=Hfaybaa@4640@, where V0⊥
 MathType@MTEF@5@5@+=feaafiart1ev1aaatCvAUfKttLearuWrP9MDH5MBPbIqV92AaeXatLxBI9gBaebbnrfifHhDYfgasaacH8akY=wiFfYdH8Gipec8Eeeu0xXdbba9frFj0=OqFfea0dXdd9vqai=hGuQ8kuc9pgc9s8qqaq=dirpe0xb9q8qiLsFr0=vr0=vr0dc8meaabaqaciaacaGaaeqabaqabeGadaaakeaat0uy0HwzTfgDPnwy1egaryqtHrhAL1wy0L2yHvdaiqaacqWFveVvdaqhaaWcbaGaeGimaadabaWeuvgwd10u51MyVXgarCWyTnxEXadaiuaacqGFLkYpaaaaaa@415B@ denotes the orthogonal complement of V
 MathType@MTEF@5@5@+=feaafiart1ev1aaatCvAUfKttLearuWrP9MDH5MBPbIqV92AaeXatLxBI9gBaebbnrfifHhDYfgasaacH8akY=wiFfYdH8Gipec8Eeeu0xXdbba9frFj0=OqFfea0dXdd9vqai=hGuQ8kuc9pgc9s8qqaq=dirpe0xb9q8qiLsFr0=vr0=vr0dc8meaabaqaciaacaGaaeqabaqabeGadaaakeaat0uy0HwzTfgDPnwy1egaryqtHrhAL1wy0L2yHvdaiqaacqWFveVvaaa@384A@_0 _in *χ*. Standard properties of the normal distribution gives

**Z**|*c *~ N(***μ***_*z*_, *c*Σ_*z*_),

where ***μ***_*z *_= *D*_*z*_***γ ***with D_*z *_= PV0⊥D
 MathType@MTEF@5@5@+=feaafiart1ev1aaatCvAUfKttLearuWrP9MDH5MBPbIqV92AaeXatLxBI9gBaebbnrfifHhDYfgasaacH8akY=wiFfYdH8Gipec8Eeeu0xXdbba9frFj0=OqFfea0dXdd9vqai=hGuQ8kuc9pgc9s8qqaq=dirpe0xb9q8qiLsFr0=vr0=vr0dc8meaabaqaciaacaGaaeqabaqabeGadaaakeaacqqGqbaudaWgaaWcbaWenfgDOvwBHrxAJfwnHbqeg0uy0HwzTfgDPnwy1aaceaGae8xfXB1aa0baaWqaaiabicdaWaqaambvLH1qnnvETj2BSbqehmwBZLxmWaacfaGae4xPI8daaaWcbeaakiabdseaebaa@43D5@, and where Σz=PV0⊥ΣPV0⊥T
 MathType@MTEF@5@5@+=feaafiart1ev1aaatCvAUfKttLearuWrP9MDH5MBPbIqV92AaeXatLxBI9gBaebbnrfifHhDYfgasaacH8akY=wiFfYdH8Gipec8Eeeu0xXdbba9frFj0=OqFfea0dXdd9vqai=hGuQ8kuc9pgc9s8qqaq=dirpe0xb9q8qiLsFr0=vr0=vr0dc8meaabaqaciaacaGaaeqabaqabeGadaaakeaacqqHJoWudaWgaaWcbaGaemOEaOhabeaakiabg2da9iabbcfaqnaaBaaaleaat0uy0HwzTfgDPnwy1egaryqtHrhAL1wy0L2yHvdaiqaacqWFveVvdaqhaaadbaGaeGimaadabaWeuvgwd10u51MyVXgarCWyTnxEXadaiuaacqGFLkYpaaaaleqaaOGaeu4OdmLaeeiuaa1aa0baaSqaaiab=vr8wnaaDaaameaacqaIWaamaeaacqGFLkYpaaaaleaacqqGubavaaaaaa@4F9A@.

The likelihood function of ***μ***_*z *_(with respect to the Lebesgue measure on the space of possible values of **Z **spanned by the column vectors of Σ_*z*_) can be written as

L(μ|Z)∝[‖Z−μz‖Σz2/2+1]−n/2−α.
 MathType@MTEF@5@5@+=feaafiart1ev1aaatCvAUfKttLearuWrP9MDH5MBPbIqV92AaeXatLxBI9gBaebbnrfifHhDYfgasaacH8akY=wiFfYdH8Gipec8Eeeu0xXdbba9frFj0=OqFfea0dXdd9vqai=hGuQ8kuc9pgc9s8qqaq=dirpe0xb9q8qiLsFr0=vr0=vr0dc8meaabaqaciaacaGaaeqabaqabeGadaaakeaacqWGmbatcqGGOaakiiWacqWF8oqBcqGG8baFieqacqGFAbGwcqGGPaqkcqGHDisTdaWadaqaamaafmaabaGae4NwaOLaeyOeI0Iae8hVd02aaSbaaSqaaiabdQha6bqabaaakiaawMa7caGLkWoadaqhaaWcbaGaeu4Odm1aaSbaaWqaaiabdQha6bqabaaaleaacqaIYaGmaaGccqGGVaWlcqaIYaGmcqGHRaWkcqaIXaqmaiaawUfacaGLDbaadaahaaWcbeqaaiabgkHiTiabd6gaUjabc+caViabikdaYiabgkHiTGGaciab9f7aHbaakiabc6caUaaa@5003@

Since, ***δ ***is estimable based on **Z**, the likelihood of ***μ***_*z *_can be examined instead of the likelihood of ***δ***.

The likelihood ratio statistic of (9) based on **X **is defined by

T=sup⁡μ∈VL(μ|X)sup⁡μ∈V0L(μ|X),
 MathType@MTEF@5@5@+=feaafiart1ev1aaatCvAUfKttLearuWrP9MDH5MBPbIqV92AaeXatLxBI9gBaebbnrfifHhDYfgasaacH8akY=wiFfYdH8Gipec8Eeeu0xXdbba9frFj0=OqFfea0dXdd9vqai=hGuQ8kuc9pgc9s8qqaq=dirpe0xb9q8qiLsFr0=vr0=vr0dc8meaabaqaciaacaGaaeqabaqabeGadaaakeaacqWGubavcqGH9aqpdaWcaaqaamaaxababaGagi4CamNaeiyDauNaeiiCaahaleaaiiWacqWF8oqBcqGHiiIZt0uy0HwzTfgDPnwy1egaryqtHrhAL1wy0L2yHvdaiqaacqGFveVvaeqaaOGaemitaWKaeiikaGIae8hVd0MaeiiFaWhcbeGae0hwaGLaeiykaKcabaWaaCbeaeaacyGGZbWCcqGG1bqDcqGGWbaCaSqaaiab=X7aTjabgIGiolab+vr8wnaaBaaameaacqaIWaamaeqaaaWcbeaakiabdYeamjabcIcaOiab=X7aTjabcYha8jab9HfayjabcMcaPaaacqGGSaalaaa@5C7F@

which can be rewritten (cf. [3]) as a strictly increasing function of

T′=n−p+2αk‖PVX−PV0X‖Σ2‖X−PVX‖Σ2+2=n−p+2αk‖PV∩V0⊥X‖Σ2‖PV⊥X‖Σ2+2,
 MathType@MTEF@5@5@+=feaafiart1ev1aaatCvAUfKttLearuWrP9MDH5MBPbIqV92AaeXatLxBI9gBaebbnrfifHhDYfgasaacH8akY=wiFfYdH8Gipec8Eeeu0xXdbba9frFj0=OqFfea0dXdd9vqai=hGuQ8kuc9pgc9s8qqaq=dirpe0xb9q8qiLsFr0=vr0=vr0dc8meaabaqaciaacaGaaeqabaqabeGadaaakeaafaqadeGabaaabaGafmivaqLbauaacqGH9aqpdaWcaaqaaiabd6gaUjabgkHiTiabdchaWjabgUcaRiabikdaYGGaciab=f7aHbqaaiabdUgaRbaadaWcaaqaamaafmaabaGaeeiuaa1aaSbaaSqaamrtHrhAL1wy0L2yHvtyaeHbnfgDOvwBHrxAJfwnaGabaiab+vr8wbqabaacbeGccqqFybawcqGHsislcqqGqbaudaWgaaWcbaGae4xfXB1aaSbaaWqaaiabicdaWaqabaaaleqaaOGae0hwaGfacaGLjWUaayPcSdWaa0baaSqaaiabfo6atbqaaiabikdaYaaaaOqaamaafmaabaGae0hwaGLaeyOeI0Iaeeiuaa1aaSbaaSqaaiab+vr8wbqabaGccqqFybawaiaawMa7caGLkWoadaqhaaWcbaGaeu4OdmfabaGaeGOmaidaaOGaey4kaSIaeGOmaidaaaqaaiabg2da9maalaaabaGaemOBa4MaeyOeI0IaemiCaaNaey4kaSIaeGOmaiJae8xSdegabaGaem4AaSgaamaalaaabaWaauWaaeaacqqGqbaudaWgaaWcbaGae4xfXBLaeyykICSae4xfXB1aa0baaWqaaiabbcdaWaqaambvLH1qnnvETj2BSbqehmwBZLxmWaacfaGaeWxPI8daaaWcbeaakiab9HfaybGaayzcSlaawQa7amaaDaaaleaacqqHJoWuaeaacqaIYaGmaaaakeaadaqbdaqaaiabbcfaqnaaBaaaleaacqGFveVvdaahaaadbeqaaiab8vQi=aaaaSqabaGccqqFybawaiaawMa7caGLkWoadaqhaaWcbaGaeu4OdmfabaGaeGOmaidaaOGaey4kaSIaeGOmaidaaiabcYcaSaaaaaa@8FB5@

where V
 MathType@MTEF@5@5@+=feaafiart1ev1aaatCvAUfKttLearuWrP9MDH5MBPbIqV92AaeXatLxBI9gBaebbnrfifHhDYfgasaacH8akY=wiFfYdH8Gipec8Eeeu0xXdbba9frFj0=OqFfea0dXdd9vqai=hGuQ8kuc9pgc9s8qqaq=dirpe0xb9q8qiLsFr0=vr0=vr0dc8meaabaqaciaacaGaaeqabaqabeGadaaakeaat0uy0HwzTfgDPnwy1egaryqtHrhAL1wy0L2yHvdaiqaacqWFveVvaaa@384A@^⊥ ^and V0⊥
 MathType@MTEF@5@5@+=feaafiart1ev1aaatCvAUfKttLearuWrP9MDH5MBPbIqV92AaeXatLxBI9gBaebbnrfifHhDYfgasaacH8akY=wiFfYdH8Gipec8Eeeu0xXdbba9frFj0=OqFfea0dXdd9vqai=hGuQ8kuc9pgc9s8qqaq=dirpe0xb9q8qiLsFr0=vr0=vr0dc8meaabaqaciaacaGaaeqabaqabeGadaaakeaat0uy0HwzTfgDPnwy1egaryqtHrhAL1wy0L2yHvdaiqaacqWFveVvdaqhaaWcbaGaeGimaadabaWeuvgwd10u51MyVXgarCWyTnxEXadaiuaacqGFLkYpaaaaaa@415B@ are the orthogonal complements of V
 MathType@MTEF@5@5@+=feaafiart1ev1aaatCvAUfKttLearuWrP9MDH5MBPbIqV92AaeXatLxBI9gBaebbnrfifHhDYfgasaacH8akY=wiFfYdH8Gipec8Eeeu0xXdbba9frFj0=OqFfea0dXdd9vqai=hGuQ8kuc9pgc9s8qqaq=dirpe0xb9q8qiLsFr0=vr0=vr0dc8meaabaqaciaacaGaaeqabaqabeGadaaakeaat0uy0HwzTfgDPnwy1egaryqtHrhAL1wy0L2yHvdaiqaacqWFveVvaaa@384A@ and V
 MathType@MTEF@5@5@+=feaafiart1ev1aaatCvAUfKttLearuWrP9MDH5MBPbIqV92AaeXatLxBI9gBaebbnrfifHhDYfgasaacH8akY=wiFfYdH8Gipec8Eeeu0xXdbba9frFj0=OqFfea0dXdd9vqai=hGuQ8kuc9pgc9s8qqaq=dirpe0xb9q8qiLsFr0=vr0=vr0dc8meaabaqaciaacaGaaeqabaqabeGadaaakeaat0uy0HwzTfgDPnwy1egaryqtHrhAL1wy0L2yHvdaiqaacqWFveVvaaa@384A@_0 _respectively.

Note that the space of possible values for ***μ***_***z ***_is V
 MathType@MTEF@5@5@+=feaafiart1ev1aaatCvAUfKttLearuWrP9MDH5MBPbIqV92AaeXatLxBI9gBaebbnrfifHhDYfgasaacH8akY=wiFfYdH8Gipec8Eeeu0xXdbba9frFj0=OqFfea0dXdd9vqai=hGuQ8kuc9pgc9s8qqaq=dirpe0xb9q8qiLsFr0=vr0=vr0dc8meaabaqaciaacaGaaeqabaqabeGadaaakeaat0uy0HwzTfgDPnwy1egaryqtHrhAL1wy0L2yHvdaiqaacqWFveVvaaa@384A@ ∩ V0⊥
 MathType@MTEF@5@5@+=feaafiart1ev1aaatCvAUfKttLearuWrP9MDH5MBPbIqV92AaeXatLxBI9gBaebbnrfifHhDYfgasaacH8akY=wiFfYdH8Gipec8Eeeu0xXdbba9frFj0=OqFfea0dXdd9vqai=hGuQ8kuc9pgc9s8qqaq=dirpe0xb9q8qiLsFr0=vr0=vr0dc8meaabaqaciaacaGaaeqabaqabeGadaaakeaat0uy0HwzTfgDPnwy1egaryqtHrhAL1wy0L2yHvdaiqaacqWFveVvdaqhaaWcbaGaeGimaadabaWeuvgwd10u51MyVXgarCWyTnxEXadaiuaacqGFLkYpaaaaaa@415B@ and that ***μ***_***z ***_= 0 under *H*_0_. Let Pz
 MathType@MTEF@5@5@+=feaafiart1ev1aaatCvAUfKttLearuWrP9MDH5MBPbIqV92AaeXatLxBI9gBaebbnrfifHhDYfgasaacH8akY=wiFfYdH8Gipec8Eeeu0xXdbba9frFj0=OqFfea0dXdd9vqai=hGuQ8kuc9pgc9s8qqaq=dirpe0xb9q8qiLsFr0=vr0=vr0dc8meaabaqaciaacaGaaeqabaqabeGadaaakeaacqqGqbaudaahaaWcbeqaaiabdQha6baaaaa@2F7D@ denote the orthogonal projection according to 〈⋅,⋅〉Σz
 MathType@MTEF@5@5@+=feaafiart1ev1aaatCvAUfKttLearuWrP9MDH5MBPbIqV92AaeXatLxBI9gBaebbnrfifHhDYfgasaacH8akY=wiFfYdH8Gipec8Eeeu0xXdbba9frFj0=OqFfea0dXdd9vqai=hGuQ8kuc9pgc9s8qqaq=dirpe0xb9q8qiLsFr0=vr0=vr0dc8meaabaqaciaacaGaaeqabaqabeGadaaakeaacqGHPms4cqGHflY1cqGGSaalcqGHflY1cqGHQms8daWgaaWcbaGaeu4Odm1aaSbaaWqaaiabdQha6bqabaaaleqaaaaa@3908@. Then, the likelihood ratio statistic of (9) based on **Z **can in analogy with (13) be shown to be a strictly increasing function of

T′z=n−p+2αk‖PV∩V0⊥zZ‖Σz2‖Z−PV∩V0⊥zZ‖Σz2+2.
 MathType@MTEF@5@5@+=feaafiart1ev1aaatCvAUfKttLearuWrP9MDH5MBPbIqV92AaeXatLxBI9gBaebbnrfifHhDYfgasaacH8akY=wiFfYdH8Gipec8Eeeu0xXdbba9frFj0=OqFfea0dXdd9vqai=hGuQ8kuc9pgc9s8qqaq=dirpe0xb9q8qiLsFr0=vr0=vr0dc8meaabaqaciaacaGaaeqabaqabeGadaaakeaacuWGubavgaqbamaaBaaaleaacqWG6bGEaeqaaOGaeyypa0ZaaSaaaeaacqWGUbGBcqGHsislcqWGWbaCcqGHRaWkcqaIYaGmiiGacqWFXoqyaeaacqWGRbWAaaWaaSaaaeaadaqbdaqaaiabbcfaqnaaDaaaleaat0uy0HwzTfgDPnwy1egaryqtHrhAL1wy0L2yHvdaiqaacqGFveVvcqGHPiYXcqGFveVvdaqhaaadbaGaeeimaadabaWeuvgwd10u51MyVXgarCWyTnxEXadaiuaacqqFLkYpaaaaleaaieGacqaF6bGEaaacbeGccqWEAbGwaiaawMa7caGLkWoadaqhaaWcbaGaeu4Odm1aaSbaaWqaaiabdQha6bqabaaaleaacqaIYaGmaaaakeaadaqbdaqaaiab7PfaAjab=jHiTiabbcfaqnaaDaaaleaacqGFveVvcqGHPiYXcqGFveVvdaqhaaadbaGaeeimaadabaGae0xPI8daaaWcbaGaeWNEaOhaaOGaeSNwaOfacaGLjWUaayPcSdWaa0baaSqaaiabfo6atnaaBaaameaacqWG6bGEaeqaaaWcbaGaeGOmaidaaOGaey4kaSIaeGOmaidaaiabc6caUaaa@7599@

The Lemma below yields that for all W⊆V0⊥
 MathType@MTEF@5@5@+=feaafiart1ev1aaatCvAUfKttLearuWrP9MDH5MBPbIqV92AaeXatLxBI9gBaebbnrfifHhDYfgasaacH8akY=wiFfYdH8Gipec8Eeeu0xXdbba9frFj0=OqFfea0dXdd9vqai=hGuQ8kuc9pgc9s8qqaq=dirpe0xb9q8qiLsFr0=vr0=vr0dc8meaabaqaciaacaGaaeqabaqabeGadaaakeaat0uy0HwzTfgDPnwy1egaryqtHrhAL1wy0L2yHvdaiqaacqWFwe=vcqGHgksZcqWFveVvdaqhaaWcbaGaeGimaadabaWeuvgwd10u51MyVXgarCWyTnxEXadaiuaacqGFLkYpaaaaaa@453F@ and all **z **∈ V0⊥
 MathType@MTEF@5@5@+=feaafiart1ev1aaatCvAUfKttLearuWrP9MDH5MBPbIqV92AaeXatLxBI9gBaebbnrfifHhDYfgasaacH8akY=wiFfYdH8Gipec8Eeeu0xXdbba9frFj0=OqFfea0dXdd9vqai=hGuQ8kuc9pgc9s8qqaq=dirpe0xb9q8qiLsFr0=vr0=vr0dc8meaabaqaciaacaGaaeqabaqabeGadaaakeaat0uy0HwzTfgDPnwy1egaryqtHrhAL1wy0L2yHvdaiqaacqWFveVvdaqhaaWcbaGaeGimaadabaWeuvgwd10u51MyVXgarCWyTnxEXadaiuaacqGFLkYpaaaaaa@415B@, ‖z‖Σz2=‖z‖Σ2
 MathType@MTEF@5@5@+=feaafiart1ev1aaatCvAUfKttLearuWrP9MDH5MBPbIqV92AaeXatLxBI9gBaebbnrfifHhDYfgasaacH8akY=wiFfYdH8Gipec8Eeeu0xXdbba9frFj0=OqFfea0dXdd9vqai=hGuQ8kuc9pgc9s8qqaq=dirpe0xb9q8qiLsFr0=vr0=vr0dc8meaabaqaciaacaGaaeqabaqabeGadaaakeaadaqbdaqaaGqabiab=Pha6bGaayzcSlaawQa7amaaDaaaleaacqqHJoWudaWgaaadbaGaemOEaOhabeaaaSqaaiabikdaYaaakiabg2da9maafmaabaGae8NEaOhacaGLjWUaayPcSdWaa0baaSqaaiabfo6atbqaaiabikdaYaaaaaa@3E01@ and PWzz=PWz
 MathType@MTEF@5@5@+=feaafiart1ev1aaatCvAUfKttLearuWrP9MDH5MBPbIqV92AaeXatLxBI9gBaebbnrfifHhDYfgasaacH8akY=wiFfYdH8Gipec8Eeeu0xXdbba9frFj0=OqFfea0dXdd9vqai=hGuQ8kuc9pgc9s8qqaq=dirpe0xb9q8qiLsFr0=vr0=vr0dc8meaabaqaciaacaGaaeqabaqabeGadaaakeaacqqGqbaudaqhaaWcbaWenfgDOvwBHrxAJfwnHbqeg0uy0HwzTfgDPnwy1aaceaGae8NfXFfabaGaemOEaOhaaGqabOGae4NEaONaeyypa0Jaeeiuaa1aaSbaaSqaaiab=zr8xbqabaGccqGF6bGEaaa@4267@. The equivalence of the test statistics (13) and (14) is now straight-forward,

T′z=n−p+2αk‖PV∩V0⊥zZ‖Σz2‖Z−vV∩V0⊥zZ‖Σz2+2=n−p+2αk‖PV∩V0⊥PV0⊥X‖Σ2‖(PV+PV⊥)(PV0⊥X−PV∩V0⊥PV0⊥X)‖Σ2+2=n−p+2αk‖PV∩V0⊥X‖Σ2‖PV⊥X‖Σ2+2=T′.
 MathType@MTEF@5@5@+=feaafiart1ev1aaatCvAUfKttLearuWrP9MDH5MBPbIqV92AaeXatLxBI9gBaebbnrfifHhDYfgasaacH8akY=wiFfYdH8Gipec8Eeeu0xXdbba9frFj0=OqFfea0dXdd9vqai=hGuQ8kuc9pgc9s8qqaq=dirpe0xb9q8qiLsFr0=vr0=vr0dc8meaabaqaciaacaGaaeqabaqabeGadaaakeaafaqaaeWadaaabaGafmivaqLbauaadaWgaaWcbaGaemOEaOhabeaaaOqaaiabg2da9aqaamaalaaabaGaemOBa4MaeyOeI0IaemiCaaNaey4kaSIaeGOmaidcciGae8xSdegabaGaem4AaSgaamaalaaabaWaauWaaeaacqqGqbaudaqhaaWcbaWenfgDOvwBHrxAJfwnHbqeg0uy0HwzTfgDPnwy1aaceaGae4xfXBLaeyykICSae4xfXB1aa0baaWqaaiabbcdaWaqaambvLH1qnnvETj2BSbqehmwBZLxmWaacfaGae0xPI8daaaWcbaGaemOEaOhaaGqabOGaeWNwaOfacaGLjWUaayPcSdWaa0baaSqaaiabfo6atnaaBaaameaacqWG6bGEaeqaaaWcbaGaeGOmaidaaaGcbaWaauWaaeaacqaFAbGwcqGHsislcqGFVeVDdaqhaaWcbaGae4xfXBLaeyykICSae4xfXB1aa0baaWqaaiabbcdaWaqaaiab9vQi=aaaaSqaaiabdQha6baakiab8PfaAbGaayzcSlaawQa7amaaDaaaleaacqqHJoWudaWgaaadbaGaemOEaOhabeaaaSqaaiabikdaYaaakiabgUcaRiabikdaYaaaaeaaaeaacqGH9aqpaeaadaWcaaqaaiabd6gaUjabgkHiTiabdchaWjabgUcaRiabikdaYiab=f7aHbqaaiabdUgaRbaadaWcaaqaamaafmaabaGaeeiuaa1aaSbaaSqaaiab+vr8wjabgMIihlab+vr8wnaaDaaameaacqaIWaamaeaacqqFLkYpaaaaleqaaOGaeeiuaa1aaSbaaSqaaiab+vr8wnaaDaaameaacqaIWaamaeaacqqFLkYpaaaaleqaaOGaeWhwaGfacaGLjWUaayPcSdWaa0baaSqaaiabfo6atbqaaiabikdaYaaaaOqaamaafmaabaGaeiikaGIaeeiuaa1aaSbaaSqaaiab+vr8wbqabaGccqGHRaWkcqqGqbaudaWgaaWcbaGae4xfXB1aaWbaaWqabeaacqqFLkYpaaaaleqaaOGaeiykaKIaeiikaGIaeeiuaa1aaSbaaSqaaiab+vr8wnaaDaaameaacqaIWaamaeaacqqFLkYpaaaaleqaaOGaeWhwaGLaeyOeI0Iaeeiuaa1aaSbaaSqaaiab+vr8wjabgMIihlab+vr8wnaaDaaameaacqaIWaamaeaacqqFLkYpaaaaleqaaOGaeeiuaa1aaSbaaSqaaiab+vr8wnaaDaaameaacqaIWaamaeaacqqFLkYpaaaaleqaaOGaeWhwaGLaeiykaKcacaGLjWUaayPcSdWaa0baaSqaaiabfo6atbqaaiabikdaYaaakiabgUcaRiabikdaYaaaaeaaaeaacqGH9aqpaeaadaWcaaqaaiabd6gaUjabgkHiTiabdchaWjabgUcaRiabikdaYiab=f7aHbqaaiabdUgaRbaadaWcaaqaamaafmaabaGaeeiuaa1aaSbaaSqaaiab+vr8wjabgMIihlab+vr8wnaaDaaameaacqaIWaamaeaacqqFLkYpaaaaleqaaOGaeWhwaGfacaGLjWUaayPcSdWaa0baaSqaaiabfo6atbqaaiabikdaYaaaaOqaamaafmaabaGaeeiuaa1aaSbaaSqaaiab+vr8wnaaCaaameqabaGae0xPI8daaaWcbeaakiab8HfaybGaayzcSlaawQa7amaaDaaaleaacqqHJoWuaeaacqaIYaGmaaGccqGHRaWkcqaIYaGmaaGaeyypa0JafmivaqLbauaacqGGUaGlaaaaaa@EDB6@

**Lemma ***Let *W
 MathType@MTEF@5@5@+=feaafiart1ev1aaatCvAUfKttLearuWrP9MDH5MBPbIqV92AaeXatLxBI9gBaebbnrfifHhDYfgasaacH8akY=wiFfYdH8Gipec8Eeeu0xXdbba9frFj0=OqFfea0dXdd9vqai=hGuQ8kuc9pgc9s8qqaq=dirpe0xb9q8qiLsFr0=vr0=vr0dc8meaabaqaciaacaGaaeqabaqabeGadaaakeaat0uy0HwzTfgDPnwy1egaryqtHrhAL1wy0L2yHvdaiqaacqWFwe=vaaa@384C@*be a subspace of χ and let *PW
 MathType@MTEF@5@5@+=feaafiart1ev1aaatCvAUfKttLearuWrP9MDH5MBPbIqV92AaeXatLxBI9gBaebbnrfifHhDYfgasaacH8akY=wiFfYdH8Gipec8Eeeu0xXdbba9frFj0=OqFfea0dXdd9vqai=hGuQ8kuc9pgc9s8qqaq=dirpe0xb9q8qiLsFr0=vr0=vr0dc8meaabaqaciaacaGaaeqabaqabeGadaaakeaacqqGqbaudaWgaaWcbaWenfgDOvwBHrxAJfwnHbqeg0uy0HwzTfgDPnwy1aaceaGae8NfXFfabeaaaaa@399F@*be the orthogonal projection from χ onto *W
 MathType@MTEF@5@5@+=feaafiart1ev1aaatCvAUfKttLearuWrP9MDH5MBPbIqV92AaeXatLxBI9gBaebbnrfifHhDYfgasaacH8akY=wiFfYdH8Gipec8Eeeu0xXdbba9frFj0=OqFfea0dXdd9vqai=hGuQ8kuc9pgc9s8qqaq=dirpe0xb9q8qiLsFr0=vr0=vr0dc8meaabaqaciaacaGaaeqabaqabeGadaaakeaat0uy0HwzTfgDPnwy1egaryqtHrhAL1wy0L2yHvdaiqaacqWFwe=vaaa@384C@. *Then for any ***x**_1_, **x**_2 _∈ W
 MathType@MTEF@5@5@+=feaafiart1ev1aaatCvAUfKttLearuWrP9MDH5MBPbIqV92AaeXatLxBI9gBaebbnrfifHhDYfgasaacH8akY=wiFfYdH8Gipec8Eeeu0xXdbba9frFj0=OqFfea0dXdd9vqai=hGuQ8kuc9pgc9s8qqaq=dirpe0xb9q8qiLsFr0=vr0=vr0dc8meaabaqaciaacaGaaeqabaqabeGadaaakeaat0uy0HwzTfgDPnwy1egaryqtHrhAL1wy0L2yHvdaiqaacqWFwe=vaaa@384C@,

〈x1,x2〉Σ=〈x1,x2〉ΣW,
 MathType@MTEF@5@5@+=feaafiart1ev1aaatCvAUfKttLearuWrP9MDH5MBPbIqV92AaeXatLxBI9gBaebbnrfifHhDYfgasaacH8akY=wiFfYdH8Gipec8Eeeu0xXdbba9frFj0=OqFfea0dXdd9vqai=hGuQ8kuc9pgc9s8qqaq=dirpe0xb9q8qiLsFr0=vr0=vr0dc8meaabaqaciaacaGaaeqabaqabeGadaaakeaacqGHPms4ieqacqWF4baEdaWgaaWcbaGaeGymaedabeaakiabcYcaSiab=Hha4naaBaaaleaacqaIYaGmaeqaaOGaeyOkJe=aaSbaaSqaaiabfo6atbqabaGccqGH9aqpcqGHPms4cqWF4baEdaWgaaWcbaGaeGymaedabeaakiabcYcaSiab=Hha4naaBaaaleaacqaIYaGmaeqaaOGaeyOkJe=aaSbaaSqaaiabfo6atnaaBaaameaat0uy0HwzTfgDPnwy1egaryqtHrhAL1wy0L2yHvdaiqaacqGFwe=vaeqaaaWcbeaakiabcYcaSaaa@511D@

where ΣW=PWΣPW
 MathType@MTEF@5@5@+=feaafiart1ev1aaatCvAUfKttLearuWrP9MDH5MBPbIqV92AaeXatLxBI9gBaebbnrfifHhDYfgasaacH8akY=wiFfYdH8Gipec8Eeeu0xXdbba9frFj0=OqFfea0dXdd9vqai=hGuQ8kuc9pgc9s8qqaq=dirpe0xb9q8qiLsFr0=vr0=vr0dc8meaabaqaciaacaGaaeqabaqabeGadaaakeaacqqHJoWudaWgaaWcbaWenfgDOvwBHrxAJfwnHbqeg0uy0HwzTfgDPnwy1aaceaGae8NfXFfabeaakiabg2da9iabbcfaqnaaBaaaleaacqWFwe=vaeqaaOGaeu4OdmLaeeiuaa1aaSbaaSqaaiab=zr8xbqabaaaaa@4306@.

**Proof **Let *A *be a matrix of a change of basis [20] from the standard basis to an orthonormal basis of *χ *such that the first *l *basis vectors span W
 MathType@MTEF@5@5@+=feaafiart1ev1aaatCvAUfKttLearuWrP9MDH5MBPbIqV92AaeXatLxBI9gBaebbnrfifHhDYfgasaacH8akY=wiFfYdH8Gipec8Eeeu0xXdbba9frFj0=OqFfea0dXdd9vqai=hGuQ8kuc9pgc9s8qqaq=dirpe0xb9q8qiLsFr0=vr0=vr0dc8meaabaqaciaacaGaaeqabaqabeGadaaakeaat0uy0HwzTfgDPnwy1egaryqtHrhAL1wy0L2yHvdaiqaacqWFwe=vaaa@384C@. Let *I*_(*l*) _denote the identity matrix with all but the *l *top left diagonal elements set to zero. It follows that *A*^T ^*A *= Σ^-1 ^and *A*PW
 MathType@MTEF@5@5@+=feaafiart1ev1aaatCvAUfKttLearuWrP9MDH5MBPbIqV92AaeXatLxBI9gBaebbnrfifHhDYfgasaacH8akY=wiFfYdH8Gipec8Eeeu0xXdbba9frFj0=OqFfea0dXdd9vqai=hGuQ8kuc9pgc9s8qqaq=dirpe0xb9q8qiLsFr0=vr0=vr0dc8meaabaqaciaacaGaaeqabaqabeGadaaakeaacqqGqbaudaWgaaWcbaWenfgDOvwBHrxAJfwnHbqeg0uy0HwzTfgDPnwy1aaceaGae8NfXFfabeaaaaa@399F@ = *I*_(*l*)_*A *and therefore,

〈x1,x2〉Σ=x1TΣ−1x2=x1TATAPWx2=x1TAT(I(l))−Ax2=x1TAT(I(l)AΣATI(l))−Ax2=x1TAT(APWΣPWTAT)−Ax2=x1T(PWΣPWT)−x2,
 MathType@MTEF@5@5@+=feaafiart1ev1aaatCvAUfKttLearuWrP9MDH5MBPbIqV92AaeXatLxBI9gBaebbnrfifHhDYfgasaacH8akY=wiFfYdH8Gipec8Eeeu0xXdbba9frFj0=OqFfea0dXdd9vqai=hGuQ8kuc9pgc9s8qqaq=dirpe0xb9q8qiLsFr0=vr0=vr0dc8meaabaqaciaacaGaaeqabaqabeGadaaakeaafaqaaeGbdaaaaeaacqGHPms4ieqacqWF4baEdaWgaaWcbaGaeGymaedabeaakiabcYcaSiab=Hha4naaBaaaleaacqaIYaGmaeqaaOGaeyOkJe=aaSbaaSqaaiabfo6atbqabaaakeaacqGH9aqpaeaacqWF4baEdaqhaaWcbaGaeGymaedabaGaeeivaqfaaOGaeu4Odm1aaWbaaSqabeaacqGHsislcqaIXaqmaaGccqWF4baEdaWgaaWcbaGaeGOmaidabeaaaOqaaaqaaiabg2da9aqaaiab=Hha4naaDaaaleaacqaIXaqmaeaacqqGubavaaGccqWGbbqqdaahaaWcbeqaaiabbsfaubaakiabdgeabjabbcfaqnaaBaaaleaat0uy0HwzTfgDPnwy1egaryqtHrhAL1wy0L2yHvdaiqaacqGFwe=vaeqaaOGae8hEaG3aaSbaaSqaaiabikdaYaqabaaakeaaaeaacqGH9aqpaeaacqWF4baEdaqhaaWcbaGaeGymaedabaGaeeivaqfaaOGaemyqae0aaWbaaSqabeaacqqGubavaaGccqGGOaakcqWGjbqsdaWgaaWcbaGaeiikaGIaemiBaWMaeiykaKcabeaakiabcMcaPmaaCaaaleqabaGaeyOeI0caaOGaemyqaeKae8hEaG3aaSbaaSqaaiabikdaYaqabaaakeaaaeaacqGH9aqpaeaacqWF4baEdaqhaaWcbaGaeGymaedabaGaeeivaqfaaOGaemyqae0aaWbaaSqabeaacqqGubavaaGccqGGOaakcqWGjbqsdaWgaaWcbaGaeiikaGIaemiBaWMaeiykaKcabeaakiabdgeabjabfo6atjabdgeabnaaCaaaleqabaGaeeivaqfaaOGaemysaK0aaSbaaSqaaiabcIcaOiabdYgaSjabcMcaPaqabaGccqGGPaqkdaahaaWcbeqaaiabgkHiTaaakiabdgeabjab=Hha4naaBaaaleaacqaIYaGmaeqaaaGcbaaabaGaeyypa0dabaGae8hEaG3aa0baaSqaaiabigdaXaqaaiabbsfaubaakiabdgeabnaaCaaaleqabaGaeeivaqfaaOGaeiikaGIaemyqaeKaeeiuaa1aaSbaaSqaaiab+zr8xbqabaGccqqHJoWucqqGqbaudaqhaaWcbaGae4NfXFfabaGaeeivaqfaaOGaemyqae0aaWbaaSqabeaacqqGubavaaGccqGGPaqkdaahaaWcbeqaaiabgkHiTaaakiabdgeabjab=Hha4naaBaaaleaacqaIYaGmaeqaaaGcbaaabaGaeyypa0dabaGae8hEaG3aa0baaSqaaiabigdaXaqaaiabbsfaubaakiabcIcaOiabbcfaqnaaBaaaleaacqGFwe=vaeqaaOGaeu4OdmLaeeiuaa1aa0baaSqaaiab+zr8xbqaaiabbsfaubaakiabcMcaPmaaCaaaleqabaGaeyOeI0caaOGae8hEaG3aaSbaaSqaaiabikdaYaqabaGccqGGSaalaaaaaa@B789@

where the last equality uses the fact that (*AB*)^- ^= *B*^- ^*A*^-1 ^when A is invertible □.

The next step is to show that the MLE of ***δ ***when **X **is observed is identical to the MLE of ***δ ***when **Z **is observed. The former is defined by

δ^=Cγ^=Carg⁡min⁡γ‖X−Dγ‖Σ2.
 MathType@MTEF@5@5@+=feaafiart1ev1aaatCvAUfKttLearuWrP9MDH5MBPbIqV92AaeXatLxBI9gBaebbnrfifHhDYfgasaacH8akY=wiFfYdH8Gipec8Eeeu0xXdbba9frFj0=OqFfea0dXdd9vqai=hGuQ8kuc9pgc9s8qqaq=dirpe0xb9q8qiLsFr0=vr0=vr0dc8meaabaqaciaacaGaaeqabaqabeGadaaakeaaiiWacuWF0oazgaqcaiabg2da9iabdoeadjqb=n7aNzaajaGaeyypa0Jaem4qam0aaCbeaeaacyGGHbqycqGGYbGCcqGGNbWzcyGGTbqBcqGGPbqAcqGGUbGBaSqaaiab=n7aNbqabaGcdaqbdaqaaGqabiab+HfayjabgkHiTiabdseaejab=n7aNbGaayzcSlaawQa7amaaDaaaleaacqqHJoWuaeaacqaIYaGmaaGccqGGUaGlaaa@49F0@

Define G
 MathType@MTEF@5@5@+=feaafiart1ev1aaatCvAUfKttLearuWrP9MDH5MBPbIqV92AaeXatLxBI9gBaebbnrfifHhDYfgasaacH8akY=wiFfYdH8Gipec8Eeeu0xXdbba9frFj0=OqFfea0dXdd9vqai=hGuQ8kuc9pgc9s8qqaq=dirpe0xb9q8qiLsFr0=vr0=vr0dc8meaabaqaciaacaGaaeqabaqabeGadaaakeaat0uy0HwzTfgDPnwy1egaryqtHrhAL1wy0L2yHvdaiqaaliab=zq8hbaa@3837@_0 _= {***γ ***: *D**γ ***∈ V
 MathType@MTEF@5@5@+=feaafiart1ev1aaatCvAUfKttLearuWrP9MDH5MBPbIqV92AaeXatLxBI9gBaebbnrfifHhDYfgasaacH8akY=wiFfYdH8Gipec8Eeeu0xXdbba9frFj0=OqFfea0dXdd9vqai=hGuQ8kuc9pgc9s8qqaq=dirpe0xb9q8qiLsFr0=vr0=vr0dc8meaabaqaciaacaGaaeqabaqabeGadaaakeaat0uy0HwzTfgDPnwy1egaryqtHrhAL1wy0L2yHvdaiqaacqWFveVvaaa@384A@_0_} and G
 MathType@MTEF@5@5@+=feaafiart1ev1aaatCvAUfKttLearuWrP9MDH5MBPbIqV92AaeXatLxBI9gBaebbnrfifHhDYfgasaacH8akY=wiFfYdH8Gipec8Eeeu0xXdbba9frFj0=OqFfea0dXdd9vqai=hGuQ8kuc9pgc9s8qqaq=dirpe0xb9q8qiLsFr0=vr0=vr0dc8meaabaqaciaacaGaaeqabaqabeGadaaakeaat0uy0HwzTfgDPnwy1egaryqtHrhAL1wy0L2yHvdaiqaaliab=zq8hbaa@3837@_1 _= {***γ ***: *D**γ ***∈ V0⊥
 MathType@MTEF@5@5@+=feaafiart1ev1aaatCvAUfKttLearuWrP9MDH5MBPbIqV92AaeXatLxBI9gBaebbnrfifHhDYfgasaacH8akY=wiFfYdH8Gipec8Eeeu0xXdbba9frFj0=OqFfea0dXdd9vqai=hGuQ8kuc9pgc9s8qqaq=dirpe0xb9q8qiLsFr0=vr0=vr0dc8meaabaqaciaacaGaaeqabaqabeGadaaakeaat0uy0HwzTfgDPnwy1egaryqtHrhAL1wy0L2yHvdaiqaacqWFveVvdaqhaaWcbaGaeGimaadabaWeuvgwd10u51MyVXgarCWyTnxEXadaiuaacqGFLkYpaaaaaa@415B@} and note that for any ***γ ***there exist ***γ***_0 _∈ G
 MathType@MTEF@5@5@+=feaafiart1ev1aaatCvAUfKttLearuWrP9MDH5MBPbIqV92AaeXatLxBI9gBaebbnrfifHhDYfgasaacH8akY=wiFfYdH8Gipec8Eeeu0xXdbba9frFj0=OqFfea0dXdd9vqai=hGuQ8kuc9pgc9s8qqaq=dirpe0xb9q8qiLsFr0=vr0=vr0dc8meaabaqaciaacaGaaeqabaqabeGadaaakeaat0uy0HwzTfgDPnwy1egaryqtHrhAL1wy0L2yHvdaiqaaliab=zq8hbaa@3837@_0 _and ***γ***_1 _∈ G
 MathType@MTEF@5@5@+=feaafiart1ev1aaatCvAUfKttLearuWrP9MDH5MBPbIqV92AaeXatLxBI9gBaebbnrfifHhDYfgasaacH8akY=wiFfYdH8Gipec8Eeeu0xXdbba9frFj0=OqFfea0dXdd9vqai=hGuQ8kuc9pgc9s8qqaq=dirpe0xb9q8qiLsFr0=vr0=vr0dc8meaabaqaciaacaGaaeqabaqabeGadaaakeaat0uy0HwzTfgDPnwy1egaryqtHrhAL1wy0L2yHvdaiqaaliab=zq8hbaa@3837@_1 _such that ***γ ***= ***γ***_0 _+ ***γ***_1_. Thus,

δ^=Carg⁡min⁡γ0+γ1:γ0∈G0,γ1∈G1‖X−D(γ0+γ1)‖Σ2.
 MathType@MTEF@5@5@+=feaafiart1ev1aaatCvAUfKttLearuWrP9MDH5MBPbIqV92AaeXatLxBI9gBaebbnrfifHhDYfgasaacH8akY=wiFfYdH8Gipec8Eeeu0xXdbba9frFj0=OqFfea0dXdd9vqai=hGuQ8kuc9pgc9s8qqaq=dirpe0xb9q8qiLsFr0=vr0=vr0dc8meaabaqaciaacaGaaeqabaqabeGadaaakeaaiiWacuWF0oazgaqcaiabg2da9iabdoeadnaaxababaGagiyyaeMaeiOCaiNaei4zaCMagiyBa0MaeiyAaKMaeiOBa4galeaacqWFZoWzdaWgaaadbaGaeGimaadabeaaliabgUcaRiab=n7aNnaaBaaameaacqaIXaqmaeqaaSGaeiOoaOJae83SdC2aaSbaaWqaaiabicdaWaqabaWccqGHiiIZt0uy0HwzTfgDPnwy1egaryqtHrhAL1wy0L2yHvdaiqaacqGFge=rdaWgaaadbaGaeGimaadabeaaliabcYcaSiab=n7aNnaaBaaameaacqaIXaqmaeqaaSGaeyicI4Sae4NbXF0aaSbaaWqaaiabigdaXaqabaaaleqaaOWaauWaaeaaieqacqqFybawcqGHsislcqWGebarcqGGOaakcqWFZoWzdaWgaaWcbaGaeGimaadabeaakiabgUcaRiab=n7aNnaaBaaaleaacqaIXaqmaeqaaOGaeiykaKcacaGLjWUaayPcSdWaa0baaSqaaiabfo6atbqaaiabikdaYaaakiabc6caUaaa@6B7F@

Now, since PV0⊥+PV0=I
 MathType@MTEF@5@5@+=feaafiart1ev1aaatCvAUfKttLearuWrP9MDH5MBPbIqV92AaeXatLxBI9gBaebbnrfifHhDYfgasaacH8akY=wiFfYdH8Gipec8Eeeu0xXdbba9frFj0=OqFfea0dXdd9vqai=hGuQ8kuc9pgc9s8qqaq=dirpe0xb9q8qiLsFr0=vr0=vr0dc8meaabaqaciaacaGaaeqabaqabeGadaaakeaaliabbcfaqPWaaSbaaSqaamrtHrhAL1wy0L2yHvtyaeHbnfgDOvwBHrxAJfwnaGabaiab=vr8wnaaDaaameaacqaIWaamaeaatqvzynuttLxBI9gBaeXbJ12C5fdmaGqbaiab+vQi=aaaaSqabaGccqGHRaWkliabbcfaqPWaaSbaaSqaaiab=vr8wnaaBaaameaacqaIWaamaeqaaaWcbeaakiabg2da9iabdMeajbaa@4A55@,

δ^=Carg⁡min⁡γ0+γ1:γ0∈G0,γ1∈G1(‖PV0(X−D(γ0+γ1))+PV0⊥(X−D(γ0+γ1))‖Σ2)=Carg⁡min⁡γ0+γ1:γ0∈G0,γ1∈G1(‖PV0(X−Dγ0)‖Σ2+‖PV0⊥(X−Dγ1)‖Σz2),
 MathType@MTEF@5@5@+=feaafiart1ev1aaatCvAUfKttLearuWrP9MDH5MBPbIqV92AaeXatLxBI9gBaebbnrfifHhDYfgasaacH8akY=wiFfYdH8Gipec8Eeeu0xXdbba9frFj0=OqFfea0dXdd9vqai=hGuQ8kuc9pgc9s8qqaq=dirpe0xb9q8qiLsFr0=vr0=vr0dc8meaabaqaciaacaGaaeqabaqabeGadaaakeaafaqaaeGadaaabaaccmGaf8hTdqMbaKaaaeaacqGH9aqpaeaacqWGdbWqdaWfqaqaaiGbcggaHjabckhaYjabcEgaNjGbc2gaTjabcMgaPjabc6gaUbWcbaGae83SdC2aaSbaaWqaaiabicdaWaqabaWccqGHRaWkcqWFZoWzdaWgaaadbaGaeGymaedabeaaliabcQda6iab=n7aNnaaBaaameaacqaIWaamaeqaaSGaeyicI48enfgDOvwBHrxAJfwnHbqeg0uy0HwzTfgDPnwy1aaceaGae4NbXF0aaSbaaWqaaiabicdaWaqabaWccqGGSaalcqWFZoWzdaWgaaadbaGaeGymaedabeaaliabgIGiolab+zq8hnaaBaaameaacqaIXaqmaeqaaaWcbeaakmaabmaabaWaauWaaeaaliabbcfaqPWaaSbaaSqaaiab+vr8wnaaBaaameaacqaIWaamaeqaaaWcbeaakiabcIcaOGqabiab9HfayjabgkHiTiabdseaejabcIcaOiab=n7aNnaaBaaaleaacqaIWaamaeqaaOGaey4kaSIae83SdC2aaSbaaSqaaiabigdaXaqabaGccqGGPaqkcqGGPaqkcqGHRaWkliabbcfaqPWaaSbaaSqaaiabbAfawnaaDaaameaacqaIWaamaeaatqvzynuttLxBI9gBaeXbJ12C5fdmaGqbaiab8vQi=aaaaSqabaGccqGGOaakcqqFybawcqGHsislcqWGebarcqGGOaakcqWFZoWzdaWgaaWcbaGaeGimaadabeaakiabgUcaRiab=n7aNnaaBaaaleaacqaIXaqmaeqaaOGaeiykaKIaeiykaKcacaGLjWUaayPcSdWaa0baaSqaaiabfo6atbqaaiabikdaYaaaaOGaayjkaiaawMcaaaqaaaqaaiabg2da9aqaaiabdoeadnaaxababaGagiyyaeMaeiOCaiNaei4zaCMagiyBa0MaeiyAaKMaeiOBa4galeaacqWFZoWzdaWgaaadbaGaeGimaadabeaaliabgUcaRiab=n7aNnaaBaaameaacqaIXaqmaeqaaSGaeiOoaOJae83SdC2aaSbaaWqaaiabicdaWaqabaWccqGHiiIZcqGFge=rdaWgaaadbaGaeGimaadabeaaliabcYcaSiab=n7aNnaaBaaameaacqaIXaqmaeqaaSGaeyicI4Sae4NbXF0aaSbaaWqaaiabigdaXaqabaaaleqaaOWaaeWaaeaadaqbdaqaaSGaeeiuaaLcdaWgaaWcbaGae4xfXB1aaSbaaWqaaiabicdaWaqabaaaleqaaOGaeiikaGIae0hwaGLaeyOeI0IaemiraqKae83SdC2aaSbaaSqaaiabicdaWaqabaGccqGGPaqkaiaawMa7caGLkWoadaqhaaWcbaGaeu4OdmfabaGaeGOmaidaaOGaey4kaSYaauWaaeaaliabbcfaqPWaaSbaaSqaaiab+vr8wnaaDaaameaacqaIWaamaeaacqaFLkYpaaaaleqaaOGaeiikaGIae0hwaGLaeyOeI0IaemiraqKae83SdC2aaSbaaSqaaiabigdaXaqabaGccqGGPaqkaiaawMa7caGLkWoadaqhaaWcbaGaeu4Odm1aaSbaaWqaaiabdQha6bqabaaaleaacqaIYaGmaaaakiaawIcacaGLPaaacqGGSaalaaaaaa@D7C0@

where the second equality follows from the generalised Theorem of Pythagoras [20], the Lemma, and the fact that PV0⊥Dγ0=0
 MathType@MTEF@5@5@+=feaafiart1ev1aaatCvAUfKttLearuWrP9MDH5MBPbIqV92AaeXatLxBI9gBaebbnrfifHhDYfgasaacH8akY=wiFfYdH8Gipec8Eeeu0xXdbba9frFj0=OqFfea0dXdd9vqai=hGuQ8kuc9pgc9s8qqaq=dirpe0xb9q8qiLsFr0=vr0=vr0dc8meaabaqaciaacaGaaeqabaqabeGadaaakeaaliabbcfaqPWaaSbaaSqaamrtHrhAL1wy0L2yHvtyaeHbnfgDOvwBHrxAJfwnaGabaiab=vr8wnaaDaaameaacqaIWaamaeaatqvzynuttLxBI9gBaeXbJ12C5fdmaGqbaiab+vQi=aaaaSqabaGccqWGebariiWacqqFZoWzdaWgaaWcbaGaeGimaadabeaakiabg2da9iabicdaWaaa@48AF@ and PV0Dγ1=0
 MathType@MTEF@5@5@+=feaafiart1ev1aaatCvAUfKttLearuWrP9MDH5MBPbIqV92AaeXatLxBI9gBaebbnrfifHhDYfgasaacH8akY=wiFfYdH8Gipec8Eeeu0xXdbba9frFj0=OqFfea0dXdd9vqai=hGuQ8kuc9pgc9s8qqaq=dirpe0xb9q8qiLsFr0=vr0=vr0dc8meaabaqaciaacaGaaeqabaqabeGadaaakeaaliabbcfaqPWaaSbaaSqaamrtHrhAL1wy0L2yHvtyaeHbnfgDOvwBHrxAJfwnaGabaiab=vr8wnaaBaaameaacqaIWaamaeqaaaWcbeaakiabdseaeHGadiab+n7aNnaaBaaaleaacqaIXaqmaeqaaOGaeyypa0JaeGimaadaaa@40BB@. Now since ***γ***_0 _and ***γ***_1 _minimise the expression independently of each other and since *C****γ***_0 _= 0 by construction,

δ^=C(arg⁡min⁡γ0∈G0‖PV0(X−Dγ0)‖Σ2+arg⁡min⁡γ1∈G1‖Z−Dzγ1‖Σz2)=Carg⁡min⁡γ1∈G1‖Z−Dzγ1‖Σz2.
 MathType@MTEF@5@5@+=feaafiart1ev1aaatCvAUfKttLearuWrP9MDH5MBPbIqV92AaeXatLxBI9gBaebbnrfifHhDYfgasaacH8akY=wiFfYdH8Gipec8Eeeu0xXdbba9frFj0=OqFfea0dXdd9vqai=hGuQ8kuc9pgc9s8qqaq=dirpe0xb9q8qiLsFr0=vr0=vr0dc8meaabaqaciaacaGaaeqabaqabeGadaaakeaafaqaaeGadaaabaaccmGaf8hTdqMbaKaaaeaacqGH9aqpaeaacqWGdbWqdaqadaqaamaaxababaGagiyyaeMaeiOCaiNaei4zaCMagiyBa0MaeiyAaKMaeiOBa4galeaacqWFZoWzdaWgaaadbaGaeGimaadabeaaliabgIGioprtHrhAL1wy0L2yHvtyaeHbnfgDOvwBHrxAJfwnaGabaiab+zq8hnaaBaaameaacqaIWaamaeqaaaWcbeaakmaafmaabaWccqqGqbaukmaaBaaaleaacqGFveVvdaWgaaadbaGaeGimaadabeaaaSqabaGccqGGOaakieqacqqFybawcqGHsislcqWGebarcqWFZoWzdaWgaaWcbaGaeGimaadabeaakiabcMcaPaGaayzcSlaawQa7amaaDaaaleaacqqHJoWuaeaacqaIYaGmaaGccqGHRaWkdaWfqaqaaiGbcggaHjabckhaYjabcEgaNjGbc2gaTjabcMgaPjabc6gaUbWcbaGae83SdC2aaSbaaWqaaiabigdaXaqabaWccqGHiiIZcqGFge=rdaWgaaadbaGaeGymaedabeaaaSqabaGcdaqbdaqaaiab9PfaAjabgkHiTiabdseaenaaBaaaleaacqWG6bGEaeqaaOGae83SdC2aaSbaaSqaaiabigdaXaqabaaakiaawMa7caGLkWoadaqhaaWcbaGaeu4Odm1aaSbaaWqaaiabdQha6bqabaaaleaacqaIYaGmaaaakiaawIcacaGLPaaaaeaaaeaacqGH9aqpaeaacqWGdbWqdaWfqaqaaiGbcggaHjabckhaYjabcEgaNjGbc2gaTjabcMgaPjabc6gaUbWcbaGae83SdC2aaSbaaWqaaiabigdaXaqabaWccqGHiiIZcqGFge=rdaWgaaadbaGaeGymaedabeaaaSqabaGcdaqbdaqaaiab9PfaAjabgkHiTiabdseaenaaBaaaleaacqWG6bGEaeqaaOGae83SdC2aaSbaaSqaaiabigdaXaqabaaakiaawMa7caGLkWoadaqhaaWcbaGaeu4Odm1aaSbaaWqaaiabdQha6bqabaaaleaacqaIYaGmaaGccqGGUaGlaaaaaa@9F1F@

For all ***γ***_0 _∈ G
 MathType@MTEF@5@5@+=feaafiart1ev1aaatCvAUfKttLearuWrP9MDH5MBPbIqV92AaeXatLxBI9gBaebbnrfifHhDYfgasaacH8akY=wiFfYdH8Gipec8Eeeu0xXdbba9frFj0=OqFfea0dXdd9vqai=hGuQ8kuc9pgc9s8qqaq=dirpe0xb9q8qiLsFr0=vr0=vr0dc8meaabaqaciaacaGaaeqabaqabeGadaaakeaat0uy0HwzTfgDPnwy1egaryqtHrhAL1wy0L2yHvdaiqaaliab=zq8hbaa@3837@_0_, *C****γ***_0 _= 0 and *D*_*z*_***γ***_0 _= 0, so the area of minimisation can be extended,

δ^=Carg⁡min⁡γ‖Z−Dzγ‖Σz2,
 MathType@MTEF@5@5@+=feaafiart1ev1aaatCvAUfKttLearuWrP9MDH5MBPbIqV92AaeXatLxBI9gBaebbnrfifHhDYfgasaacH8akY=wiFfYdH8Gipec8Eeeu0xXdbba9frFj0=OqFfea0dXdd9vqai=hGuQ8kuc9pgc9s8qqaq=dirpe0xb9q8qiLsFr0=vr0=vr0dc8meaabaqaciaacaGaaeqabaqabeGadaaakeaaiiWacuWF0oazgaqcaiabg2da9iabdoeadnaaxababaGagiyyaeMaeiOCaiNaei4zaCMagiyBa0MaeiyAaKMaeiOBa4galeaacqWFZoWzaeqaaOWaauWaaeaaieqacqGFAbGwcqGHsislcqWGebardaWgaaWcbaGaemOEaOhabeaakiab=n7aNbGaayzcSlaawQa7amaaDaaaleaacqqHJoWudaWgaaadbaGaemOEaOhabeaaaSqaaiabikdaYaaakiabcYcaSaaa@4991@

which is the MLE of ***δ ***based on **Z **by definition.    □

**Remark 1 **Using the invertible map between any two choices of **Y**, **Y **and **Y'**, as defined in Step 1 above, the respective maximum likelihood estimates of *α*, Σ_*y *_and Σ_*y' *_can be shown to be identical based on **Y **or **Y'**. In this sense, the choice of μ˜0
 MathType@MTEF@5@5@+=feaafiart1ev1aaatCvAUfKttLearuWrP9MDH5MBPbIqV92AaeXatLxBI9gBaebbnrfifHhDYfgasaacH8akY=wiFfYdH8Gipec8Eeeu0xXdbba9frFj0=OqFfea0dXdd9vqai=hGuQ8kuc9pgc9s8qqaq=dirpe0xb9q8qiLsFr0=vr0=vr0dc8meaabaqaciaacaGaaeqabaqabeGadaaakeaaiiWacuWF8oqBgaacamaaCaaaleqabaGaeGimaadaaaaa@2F94@ is thus truly irrelevant.

**Remark 2 **Sometimes, additional linear combinations of ***γ ***can be assumed to be zero for most genes, C* ***γ ***= 0 for some matrix C* with rowspace being a superspace of the rowspace of *C*. Let P* be any projection matrix on the corresponding space V
 MathType@MTEF@5@5@+=feaafiart1ev1aaatCvAUfKttLearuWrP9MDH5MBPbIqV92AaeXatLxBI9gBaebbnrfifHhDYfgasaacH8akY=wiFfYdH8Gipec8Eeeu0xXdbba9frFj0=OqFfea0dXdd9vqai=hGuQ8kuc9pgc9s8qqaq=dirpe0xb9q8qiLsFr0=vr0=vr0dc8meaabaqaciaacaGaaeqabaqabeGadaaakeaat0uy0HwzTfgDPnwy1egaryqtHrhAL1wy0L2yHvdaiqaacqWFveVvaaa@384A@_* _= {***μ ***: ***μ ***= *D****γ***, C* ***γ ***= **0**, ***γ ***∈ ℝ^*q*^} and let Y* = **X **- P* **X**. It is straight forward to show that a variant of the Proposition still holds, so given the covariance structure matrices the inference results concerning *C**γ ***will be identical, based on **Y **or Y* respectively. However, the estimates of the covariance structure matrices for **Y **and Y* might not be coherent and the results are expected to differ slightly.

### The estimator of power

Consider a certain experimental design, a level 1-*λ *test and a differential expression *δ*. Let a realisation of the experiment be given, which e.g. results in certain quality deviations between arrays. The conditional power is defined as the probability of identifying a random gene in the current experiment, i.e. conditional on e.g. the quality deviations, when the gene has differential expression *δ*. The power is then defined as the average conditional power over all possible realisations of the experimental design. The power is thus related to an unperformed experiment while the conditional power is related to a specific performed experiment. Here, the test is assumed to be valid conditional on the experiment, i.e. to have conditional power *λ *when ***δ ***= **0**.

In Evaluation of power, the aim is to estimate the power for a hypothetical experiment where the distribution of the data under the null hypothesis is obtained by resampling of real data. For a given resample, a constant differential expression is added to randomly selected genes and the statistics *t*_*g *_are computed. The estimate  of the conditional critical value is computed so that a proportion *λ *of the unregulated genes satisfy |*t*_*g*_| ≥ . The conditional power is then estimated by the proportion of regulated genes satisfying |*t*_*g*_| ≥ . The power is finally estimated by averaging the estimated conditional power over the resamples.

## Competing interests

The author(s) declares that there are no competing interests.

## Authors' contributions

ON provided initial ideas related to the generalisation. AS formulated the generalisation, performed the proofs in Methods, designed and programmed the analyses, simulations and plots. EK and ON helped refining the generalisation and Methods. AS, EK and MR drafted the manuscript. All authors continuously provided feedback on various parts of the work leading to the manuscript and approved the final version of the manuscript.

## Supplementary Material

Additional file 1 **Pairwise plots of all arrays in the Atrium dataset**. Transformed expression values for all arrays in the Atrium dataset. See legend of Figure 2 for details. Click here for file

Additional file 2 **Pairwise plots of all arrays in the COPD dataset**. Transformed expression values for all arrays in the COPD dataset. See legend of Figure 2 for details. Click here for file

Additional file 3 **Probability plots for the Atrium dataset**. Empirical distributions of p-values for LIMMA, weighted LIMMA, OLM and WAME from tests on 100 resamples from the Atrium dataset. Average empirical distribution indicated. Since no signal is added, the curves should ideally follow the diagonal. Click here for file
