# Supplementary figures and images for: Weighted analysis of general microarray experiments
Source: BMC Bioinformatics. 2007 Oct 15;8:387. doi: 10.1186/1471-2105-8-387 (PMC2175522; doi:10.1186/1471-2105-8-387)

**LIMMA (Atrium)**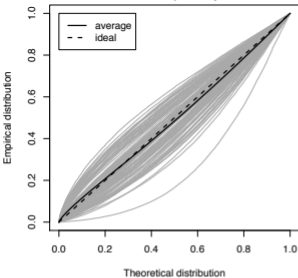**Weighted LIMMA (Atrium)**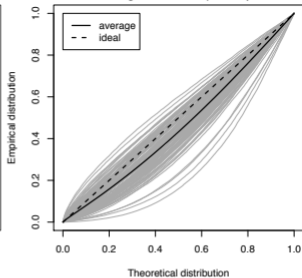**Ordinary Linear Model (Atrium)**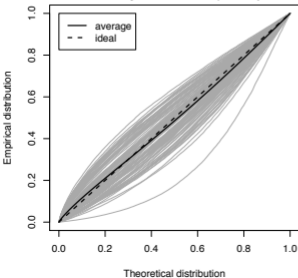**WAME (Atrium)**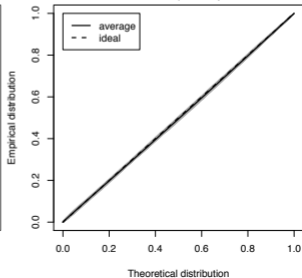

Supplement: Additional file 3 — Probability plots for the Atrium dataset. Empirical distributions of p-values for LIMMA, weighted LIMMA, OLM and WAME from tests on 100 resamples from the Atrium dataset. Average empirical distribution indicated. Since no signal is added, the curves should ideally follow the diagonal. [file 1471-2105-8-387-S3.pdf]
